# Forecasting Atherosclerotic Cardiovascular Disease in South Asia Until 2040: A Bayesian Modeling Approach

**DOI:** 10.1016/j.jacasi.2025.06.014

**Published:** 2025-08-21

**Authors:** Antoinette Cotton, Pedro RVO Salerno, Zhuo Chen, Salim Virani, Naveed Sattar, Sanjay Rajagopalan, Salil V. Deo

**Affiliations:** a Case Western Reserve University School of Medicine, Case Western Reserve University, Cleveland, Ohio, USA; b NYC + Elmhurst Hospitals, Queens, New York, USA; c Harrington Heart and Vascular Institute, University Hospitals Cleveland Medical Center, Cleveland, Ohio, USA; d Aga Khan University, Karachi, Pakistan; e Institute of Cardiovascular and Metabolic Sciences, University of Glasgow, Glasgow, Scotland, United Kingdom; f School of Health and Wellbeing, University of Glasgow, Glasgow, Scotland, United Kingdom; g Surgical Services, Louis Stokes Veteran Affairs Cleveland, Cleveland, Ohio, USA

**Keywords:** atherosclerotic cardiovascular disease, Bayesian age period cohort models, cerebrovascular disease, global burden of disease, ischemic heart disease, peripheral artery disease, South Asia

## Abstract

**Background:**

Atherosclerotic cardiovascular disease (ASCVD) disproportionately impacts low-middle income countries, such as those in South Asia and understanding future ASCVD rates can inform public policy.

**Objectives:**

This study aimed to project the burden of ASCVD in South Asia till 2040.

**Methods:**

Yearly ischemic heart disease (IHD), stroke, and peripheral artery disease (PAD) counts for South Asia (Afghanistan, Bangladesh, Bhutan, India, the Maldives, Nepal, Pakistan, and Sri Lanka) and mid-year population were obtained from Global Burden of Disease (1990-2021) in 5-year age brackets (40-79 years) and estimated mid-year national population (2022-2040) was collected. Age-adjusted prevalence (aaPR) and mortality rate (per 100,000) were projected with Bayesian age-period-cohort models in South Asia (overall, males, and females); trends were reported as the estimated annual percent change (EAPC).

**Results:**

Between 2021 and 2040, the IHD aaPR in South Asia was projected to increase (2021: 9434.6 [95% CI: 9,432.1-9,437.1], 2040: 9,846.6 [95% CI: 8,800.0-10,893.3], EAPC: 0.23% [95% CI: 0.08%-0.37%]) because of increased rates among females (EAPC: 1.16%; 95% CI: 1%-1.32%). The overall IHD age-adjusted mortality rate will reduce (2021: 254.7 [95% CI: 254.3-255.1), 2040: 224.0 [95% CI: 166.5-281.6), EAPC: −0.67% [95% CI: −1.61% to 0.27%]) but may increase in females (EAPC: 1.16%; 95% CI: 1%-1.32%). Stoke aaPR in South Asia is projected to increase slightly (2021: 1,065.5 [95% CI: 1,064.7-1,066.4], 2040: 1,074.6 [95% CI: 953.7-1,195.5]). The PAD aaPR is projected to increase (2021: 1809.5 [95% CI: 1,808.5-1,810.6], 2040: 1,879.5 [95% CI: 1,684.9-2,074.0], EAPC: 0.26% [95% CI: 0.04%-0.47%]) because of increased rates in females (EAPC: 0.29%; 95% CI: −0.01% to 0.59%).

**Conclusions:**

IHD and PAD prevalence rates are projected to increase in South Asia with a disproportionate increase among females.

Atherosclerotic cardiovascular disease (ASCVD) comprising of ischemic heart disease (IHD), stroke, and peripheral arterial disease (PAD) continues to be a leading cause of death worldwide.^1^ Although South Asia, comprising Afghanistan, Bangladesh, Bhutan, India, the Maldives, Nepal, Pakistan, and Sri Lanka, represents 25% of the global population, they presently account for 60% of the global burden of cardiovascular disease (CVD). The high prevalence of CVD is attributed to many factors including a rapid increase in cardiometabolic risk factors that include increased rates of hypertension, diabetes, and lipid disorders concomitant with the increase in many adverse environmental factors such as exposure to air pollution and chemicals and multiple social determinants of health.^2^, ^3^, ^4^, ^5^, ^6^, ^7^ The rapidly shifting landscape of risk factors attributed to urbanization in low- and middle-income countries (LMIC), including profound alterations in diet, physical activity, and emerging exposures pose a challenge for accurate future estimations of actual prevalence of ASCVD. High rates of inequality of income in many South Asian countries also represents a barrier to access to timely therapeutic interventions that could be lifesaving.^8^ Not surprisingly, ASCVD poses a substantial burden to the vulnerable economies of LMICs with a cost as far back as 2015 of U.S. $1.96 billion, U.S. $0.21 billion, and U.S. $0.14 billion, for India, Pakistan, and Bangladesh, respectively.^9^ Given the significant personal and societal burden, accurate forecasting of ASCVD is of profound significance in these countries. This data, we believe, could be used to inform future health care policy and better inform priorities in the future.

In this work, we linked data from the Global Burden of Disease (GBD) with prior and future projected mid-year population estimates to project the burden of ASCVD in South Asia.

## Methods

### Data sources

The data sources for our study were the GBD 2021 and the Shared Socioeconomic Pathway Public Database. The GBD is a study of nearly 12,000 collaborators from 163 countries and territories that aims to measure disability and death from numerous causes worldwide. In full compliance with Guidelines for Accurate and Transparent Health Estimates Reporting, the 2021 GBD provided publicly available reports of disease burden, customizable by disease of interest, location, time frame, age, and sex.^10^^,^^11^

To obtain annual burden of IHD, stroke, and PAD, mortality and prevalence counts from GBD 2021 were collected in 5-year age brackets (from 40-79 years of age) between 1990 and 2021. Angina, myocardial infarction, and ischemic cardiomyopathy were defined as IHD; rapidly developing clinical signs of disturbance of cerebral function lasting longer than 24 hours or leading to death were defined as stroke; an ankle brachial index <0.9 with symptoms of intermittent claudication were defined as PAD.^12^, ^13^, ^14^ Mortality and prevalence data obtained for each country were further processed to ensure that national and regional results could be compared.^15^ These data were collected for the overall population, and then separately for males and females in each country.

The mid-year population estimates (overall, males, and females) in the studied 5-year age brackets for the years 1990 to 2021 were obtained from GBD 2021. The mid-year projected population estimates (overall, males, and females) in each 5-year age bracket for each country were obtained from the Shared Socioeconomic Pathways Public Database. ^16^

The Shared Socioeconomic Pathways, created by the International Institute for Applied Systems Analysis and the National Center for Atmospheric Research, provides projected global population, gross domestic product, climate change, and other estimates for the remainder of the century.^17^ The population database is comprised of 5 possible scenarios, each with its own population estimates that are based on broad societal tendencies such as socioeconomic, technological, cultural, and political trends, as well as factors such as education level and fertility rate.^17^^,^^18^ The Shared Socioeconomic Pathways − 2 scenario data were chosen for our study as it reports the average projected scenario.^17^^,^^18^

### Statistical analysis

Using data from the number of occurred events and mid-year population during the observed period (1990-2021), the age-standardized mortality rates (per 100,000) and prevalence rates for IHD, stroke, and PAD were calculated by the direct standardization method. The mid-year 1990 population for each country was selected as the index for calculating the age-standardized rates. Data from individual countries were pooled to report estimates for the whole of South Asia.

Bayesian age-period-cohort (APC) models were then fitted to this observed data (1990-2021) to project age-standardized rates (with the mid-year 1990 population as the index) for the prevalence and mortality for IHD, stroke, and PAD from 2022 through 2040. The Bayesian APC models were fitted using a Poisson distribution and a log link. All 3 terms (age, period, and cohort) were included simultaneously in the model. Adding age, period, and cohort models in routine regression analyses is not possible as each can be exactly derived from the other 2 terms. However, this issue (identifiability) is resolved in the Bayesian APC approach by adding small perturbations to each term before fitting models. Default log-normal priors were chosen for each term in the model. The posterior estimates from the model fit were obtained using the integrated network Laplace approximation method. As projections from Bayesian APC models are prone to unduly large variance estimates, results for these models were reported by obtaining the median, 25^th^ percentile, and 75^th^ percentile values from the model posterior distributions. Further details regarding Bayesian APC models are provided in the Supplemental Material.

Additionally, the temporal change for the observed (1990-2021) and projected periods (2022-2040) was reported using the estimated annual percentage change (EAPC) with the 95% CI calculated using bootstrap simulations. Results of EAPC where the 95% CI does not subtend 0 are statistically significant with a *P* < 0.05 at the 95% CI.

The above analyses were performed for the whole nation (overall), and separately for males and females. R 4.3.2 (R Foundation for Statistical Computing) was used for all statistical analyses; PHEindicatormethods^19^ and BAPC^20^ were the main packages used for modeling. As the data used are publicly available, the study was exempted from institutional review board approval as the data are at the population level; the study was also exempted from needing individual patient consent. Code used in the study can be requested as a single zip file from the corresponding author. The data can be obtained directly from the GBD portal.

## Results

### Population trends

Between 2021 and 2040, the population of South Asia is expected to grow by 56.6% in the 40- to 79-year age bracket. Similarly, the population for all constituent countries is also projected to increase during the same period. The smallest relative increase is expected in Sri Lanka (15.2%) whereas the largest increase in expected in Afghanistan (178.2%) (Supplemental Table 1).

### IHD Prevalence

**Figure 1:**
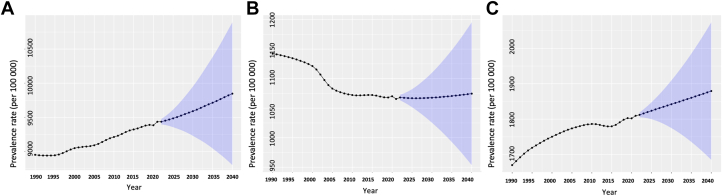
Age-Standardized ASCVD Prevalence Rates in South Asia The Global Burden of Disease data and future mid-year population estimates are modelled to project the population-level age-standardized prevalence and mortality rates for South Asia (2022-2040). Shown are the observed (1990-2021) and the projected (2022-2040) prevalence of (A) ischemic heart disease, (B) stroke, and (C) peripheral artery disease. ASCVD = atherosclerotic cardiovascular disease.

**Figure 2:**
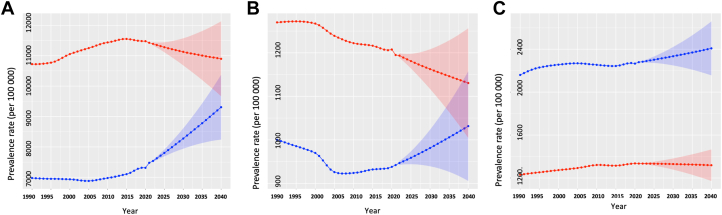
Sex-Stratified Age-Standardized ASCVD Prevalence Rates in South Asia The Global Burden of Disease data and future mid-year population estimates were modelled to project the population-level age-standardized prevalence and mortality rates for South Asia (2022-2040). Shown are the observed (1990-2021) and the projected (2022-2040) age-standardized prevalence rates of (A) ischemic heart disease, (B) stroke, and (C) peripheral artery disease for males and females. The red and blue color depict results for males and females respectively. Abbreviation as in Figure 1.

For South Asia, the age-standardized IHD prevalence rate increased between 1990 and 2021 (1990: 8,951.1 [95% CI: 8,947.2-8,955.0]; 2021: 9,434.6 [95% CI: 9,432.1-9,437.1]; EAPC: 0.17% [95% CI: 0.08%-0.26%]). Although the age-standardized IHD prevalence rate was observed to decline in Afghanistan (EAPC: −0.29% [95% CI: −0.36% to −0.22%]) and Maldives (EAPC: −0.21% [95% CI: −0.35% to −0.07%]), it increased in India (EAPC: 0.23% [95% CI: 0.13%-0.32%]) (Supplemental Table 2, Figure 1A). The age-standardized prevalence rate in South Asia was observed to increase equally among males (EAPC: 0.21%; 95% CI: 0.12%-0.29%) and females (EAPC: 0.22%; 95% CI: 0.12%, 0.33%) (Figure 2A). Among constituent nations, trends for females and males were similar, with rates declining in Afghanistan and Maldives, whereas they increased in India (Supplemental Tables 3 and 4). During the projected period (2021-2040), the age-standardized IHD prevalence rate in South Asia was expected to increase further (2021: 9,434.6 [95% CI: 9,432.1-9,437.1], 2040: 9846.6 [95% CI: 8,800.0-10,893.3], EAPC: 0.23% [95% CI: 0.08%-0.37%]) (Figure 1A). Although the prevalence rate is projected to increase substantially in Afghanistan (EAPC: 0.41%; 95% CI: 0.29%-0.52%), Maldives (EAPC: 0.86%; 95% CI: 0.63%-1.09%), and India (EAPC: 0.32; 95% CI: 0.17-0.47), it is projected to decrease in Bangladesh (EAPC: −0.33%; 95% CI: −0.49% to −0.17%) (Table 1, Central Illustration). The overall increase in IHD prevalence in South Asia is primarily driven by the projected increase in females (EAPC: 1.16%; 95% CI: 1%-1.32%) as the rates among males are actually projected to decrease (EAPC: −0.25%; 95% CI: −0.39% to −0.11%) (Figure 2A). In individual countries, the age-standardized IHD prevalence for females is projected to increase in each country, whereas in males, some countries such as Bangladesh and Sri Lanka will see substantial decreases; others, such as Maldives and Nepal, will see increases in prevalence (Supplemental Tables 3 and 4).

**Table 1:** Projected Age-Standardized IHD, Stroke, and PAD Prevalence and Mortality Rates in South Asia (2021-2040)

Condition	Metric	Year	South Asia	Afghanistan	Bangladesh	Bhutan	India	Maldives	Nepal	Pakistan	Sri Lanka
IHD	Prevalence (per 100,000)	2021	9,434.6 (9,433.7-9,435.5)	15,526.9 (15,510.7-15,543.0)	8,683.3 (8,680.5-8,686.0)	8,755.3 (8,719.7-8,790.9)	9,373.0 (9,372.0-9,374.0)	3,786.9 (3,757.6-3,816.3)	7,872.5 (7,866.4-7,878.7)	11,823.3 (11,819.8-11,826.8)	4,290.6 (4,286.4-4,294.8)
		2040	9,846.6 (8,799.6-10,893.7)	16,781.1 (14,712.4-18,849.8)	8,154.2 (7,177.5-9,130.9)	9,269.5 (7,949.6-10,589.5)	9,964.6 (8,893.7-11,035.4)	4,480.7 (3,688.5-5,273.0)	8,120.0 (7,216.4-9,023.6)	11,259.8 (9,953.8-12,565.8)	4,244.4 (3,679.5-4,809.2)
	EAPC (%)	—	0.23 (0.08-0.37)	0.41 (0.29-0.52)	−0.33 (−0.49 to −0.17)	0.29 (0.14-0.45)	0.32 (0.17-0.47)	0.86 (0.63-1.09)	0.16 (0.00- 0.33)	−0.26 (−0.39 to −0.12)	−0.06 (−0.28 to 0.17)
	Mortality (per 100,000)	2021	254.7 (254.6-254.8)	547.4 (544.9-550.0)	179.4 (179.0-179.7)	158.6 (155.1-162.1)	257.2 (257.1-257.4)	85.2 (81.9-88.4)	211.2 (210.2-212.1)	328.9 (328.3-329.5)	145.9 (145.2-146.7)
		2040	224.0 (166.5-281.6)	515.1 (412.6-617.5)	142.7 (108.1-177.3)	133.7 (99.7-167.7)	230.9 (162.5-299.3)	50.3 (31.9-68.7)	189.4 (135.6-243.2)	286.7 (209.8-363.7)	123.6 (19.4-227.8)
	EAPC (%)	—	−0.67 (−1.61 to 0.27)	−0.34 (−0.97 to 0.29)	−1.19 (−2.33 to −0.04)	−0.92 (−2.11 to 0.29)	−0.57 (−1.49 to 0.37)	−2.91 (−4.67 to −1.13)	−0.57 (−1.60 to 0.46)	−0.72 (−1.55 to 0.11)	−0.90 (−2.14 to 0.35)
Stroke	Prevalence (per 100,000)	2021	1,065.6 (1,065.3-1,065.9)	2,016.6 (2,011.5- 2,021.7)	1,288.7 (1,287.6-1,289.7)	1,070.0 (1,059.2-1,080.8)	960.9 (960.6-961.2)	1,382.1 (1,365.8-1,398.5)	951.2 (949.1-953.2)	1,663.0 (1,661.7-1,664.3)	1,636.0 (1,633.2-1,638.8)
		2040	1,074.6 (953.6-1,195.5)	1,924.7 (1,682.6-2,166.7)	1,388.4 (1,211.5- 1,565.4)	1,069.0 (866.7-1,271.2)	957.5 (844.8-1,070.3)	1,197.1 (932.8-1,461.4)	932.9 (817.1-1,048.8)	1,635.8 (1,457.3-1,814.2)	1,428.9 (1,160.4-1,697.5)
	EAPC (%)	—	0.04 (−0.4 to 0.49)	−0.23 (−0.56 to 0.1)	0.39 (−0.01 to 0.79)	−0.01 (−0.46 to 0.43)	−0.02 (−0.49 to 0.45)	−0.76 (−1.16 to −0.35)	−0.10 (−0.58 to 0.37)	−0.09 (−0.45 to 0.27)	−0.71 (−1.08 to −0.34)
	Mortality (per 100,000)	2021	44.9 (44.8-44.9)	168.1 (166.8-169.5)	65.2 (65.0-65.5)	37.3 (35.8-38.8)	39.7 (39.7-39.8)	26.6 (25.0-28.3)	44.3 (44.0-44.7)	68.2 (68.0-68.5)	54.1 (53.7-54.6)
		2040	38.2 (27.8-48.5)	162.1 (126.0-198.1)	57.2 (39.7-74.7)	32.8 (21.8-43.8)	34.1 (23.3-44.9)	14.2 (8.1-20.4)	38.2 (29.5-46.9)	58.7 (43.9-73.6)	29.8 (16.4-43.1)
	EAPC (%)	—	−0.85 (−3.07 to 1.43)	−0.18 (−1.31 to 0.96)	−0.69 (−2.53 to 1.19)	−0.80 (−3.21 to 1.67)	−0.80 (−3.16 to 1.62)	−3.47 (−6.67 to −0.17)	−0.79 (−3.02 to 1.5)	−0.79 (−2.6 to 1.04)	−3.16 (−5.4 to −0.85)
PAD	Prevalence (per 100,000)	2021	1,809.5 (1,809.2-1,809.9)	2,130.3 (2,124.9-2,135.7)	1,646.0 (1,644.8-1,647.2)	1,505.1 (1,492.0-1,518.2)	1,773.5 (1,773.1-1,773.9)	2,806.3 (2,781.6-2,830.9)	1,732.5 (1,729.6−1,735.3)	2,024.4 (2,023.0-2,025.9)	3,133.3 (3,129.7-3,136.9)
		2040	1,879.5 (1,685.0-2074.0)	2,233.0 (1,941.5-2,524.5)	1,731.5 (1,535.2-1,927.8)	1,570.7 (1,287.0-1,854.5)	1,840.2 (1,643.9-2,036.6)	2,684.3 (2,193.5-3,175.2)	1,826.5 (1,579.9-2,073.1)	2,135.6 (1,887.1-2,384.1)	3,130.6 (2,767.6-3,493.6)
	EAPC (%)	—	0.20 (−0.14 to 0.54)	0.25 (−0.06 to 0.57)	0.27 (−0.09 to 0.62)	0.22 (−0.15 to 0.59)	0.19 (−0.15 to 0.54)	−0.24 (−0.51 to 0.04)	0.28 (−0.07 to 0.62)	0.28 (−0.04 to 0.6)	−0.01 (−0.27 to 0.26)

Values are rates (95% CIs).

EAPC = estimated annual percent change; IHD = ischemic heart disease; PAD = peripheral artery disease.

**Central Illustration:**
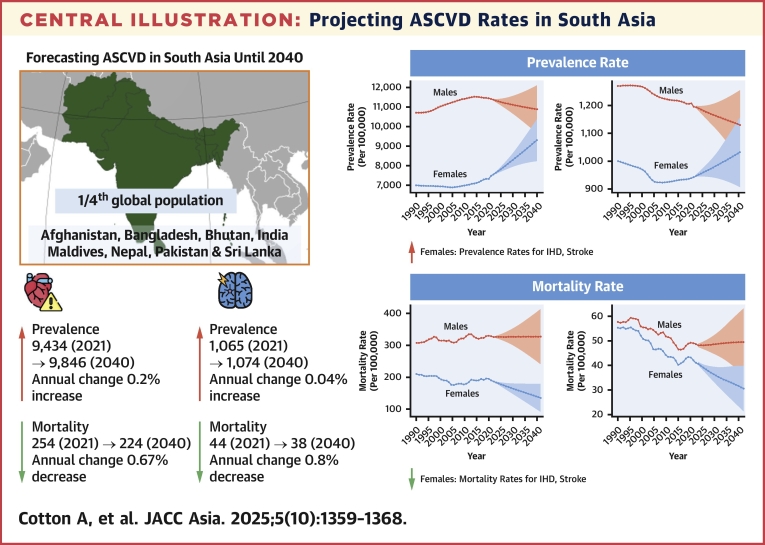
Projecting ASCVD Rates in South Asia Bayesian age-period-cohort models are applied to project the prevalence of ischemic heart disease (IHD), stroke, and peripheral artery disease (PAD) in South Asia, overall, and stratified by sex. Overall, the burden for both IHD and PAD is expected to increase in South between 2021 and 2040. However, the age-standardized IHD and stroke mortality rates are expected to decrease. ASCVD = atherosclerotic cardiovascular disease.

### IHD mortality

**Figure 3:**
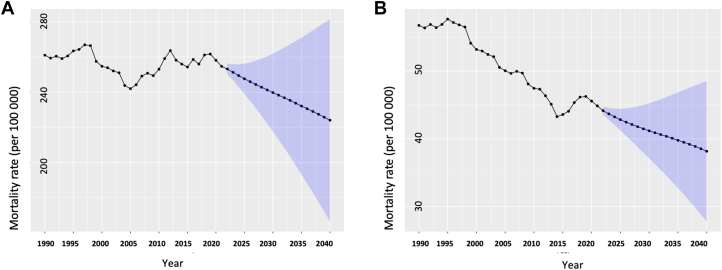
Age-Standardized ASCVD Mortality Rates in South Asia The Global Burden of Disease data and future mid-year population estimates are modelled to project the population-level age-standardized prevalence and mortality rates for South Asia (2022-2040). Shown are the observed (1990-2021) and the projected (2022-2040) mortality of (A) ischemic heart disease and (B) stroke. Abbreviation as in Figure 1.

**Figure 4:**
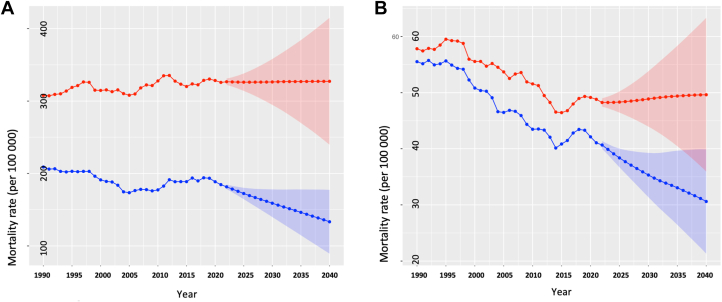
Sex-Stratified Age-Standardized ASCVD Mortality Rates in South Asia The Global Burden of Disease data and future mid-year population estimates are modelled to project the population-level age-standardized prevalence and mortality rates for South Asia (2022-2040). Shown are the observed (1990-2021) and the projected (2022-2040) age-standardized mortality rates of (A) ischemic heart disease and (B) stroke for males and females. The red and blue color depicts results for males and females, respectively. Abbreviation as in Figure 1.

The age-standardized IHD mortality rate in South Asia decreased between 1990 and 2021 (1990: 261.0 [95% CI: 260.4-261.7), 2021: 254.7 [95% CI: 254.3-255.1], EAPC: −0.08% [95% CI: −0.63% to 0.48%]) (Figure 3A). This reduction was observed in all countries expect Pakistan (EAPC: 0.71%; 95% CI: 0.18%-1.24%) where it increased during this period. The age-standardized IHD mortality rate between 1990 and 2021 also increased slightly in India (EAPC: 0.01%; 95% CI: −0.55% to 0.57%) (Supplemental Table 5). This increase in age-standardized IHD mortality was primarily due to an increase among males (EAPC: 0.18%; 95% CI: −0.32% to 0.69%) as the rate among females decreased (EAPC: −0.4%; 95% CI: −1.03% to 0.24%) (Figure 4A). Sex-specific trends varied between constituent nations (Supplemental Tables 6 and 7). During the projected period (2021-2040) the age-standardized IHD mortality rate in South Asia was expected to decrease further (2021: 254.7 [95% CI: 254.3-255.1], 2040: 224.0 [95% CI: 166.5-281.6], EAPC: −0.67% [95% CI: −1.61% to 0.27%]) (Figure 3A). This reduction in age-standardized mortality was observed in each county in South Asia (Table 1). However, the decrease in the age-standardized IHD mortality was primarily due to a reduction among males (EAPC: −0.25%; 95% CI: −0.39% to −0.11%) as the rate among females is projected to increase (EAPC: 1.16%; 95% CI: 1%-1.32%) (Figure 4A). In individual countries, IHD mortality rate in males is projected to decrease in every country except for India, whereas for females, it is projected to decrease in each country (Supplemental Tables 6 and 7).

### Stroke prevalence

The age-standardized stroke prevalence rate in South Asia decreased between 1990 and 2021 (1990: 1,142.3 [95% CI: 1,140.9-1,143.7], 2021: 1,065.5 [95% CI: 1,064.7-1,066.4], EAPC: −0.22% [95% CI: −0.49% to 0.04%]) (Figure 1B) and this trend was consistent across all nations (Supplemental Table 8). The decrease in the age-standardized stroke prevalence rate in South Asia was comparable between females (EAPC: −0.19%; 95% CI: −0.48% to 0.09%) and males (EAPC: −0.2%; 95% CI: −0.45% to 0.06%) (Figure 2B). However, trends varied between nations; the age-standardized prevalence rate for stroke in Afghanistan decreased in females but increased in males, whereas in Pakistan, the rate increased in females and decreased in males (Supplemental Tables 9 and 10). During the projected period (2021-2040), the age-standardized stroke prevalence rate in South Asia increased slightly (2021: 1,065.5 [95% CI: 1,064.7-1,066.4], 2040: 1,074.6 [95% CI: 953.7-1,195.5], EAPC: 0.04% [95% CI: −0.4% to 0.49%]) (Table 1, Figure 1B). Although the prevalence is projected to increase in Bangladesh (EAPC: 0.39%; 95% CI: −0.01% to 0.79%), it is expected to decrease in the Maldives (EAPC: −0.76%; 95% CI: −1.16% to −0.35%) and Sri Lanka (EAPC: −0.71%; 95% CI: −1.08% to −0.34%) (Table 1). The stroke prevalence rate of South Asia will decrease in males (EAPC: −0.29%; 95% CI: −0.72% to 0.14%) but increase in females (EAPC: 0.48%; 95% CI: 0.01%-0.95%) (Figure 2B). In individual countries, there is a projected decrease in stroke prevalence among males for every country except for Bangladesh (Supplemental Table 9). Projected trends in stroke prevalence in females varies; some countries, such as Bangladesh and India, will see increases in stroke prevalence for females, whereas others, such as the Maldives and Afghanistan will see decreases (Supplemental Table 10).

### Stroke mortality

The age-standardized stroke mortality rate in South Asia decreased between 1990 and 2021 (1990: 56.7 [95% CI: 56.4-57.0], 2021: 44.9 [95% CI: 44.7-45.0], EAPC: −0.75% (95% CI: −2% to 0.51%]) (Figure 3B). The age-standardized stroke prevalence rate decreased in all countries but Pakistan (EAPC: 0.08%; 95% CI: −1.01% to 1.17%) (Supplemental Table 11). During this period, the age-standardized stroke mortality rate in South Asia decreased for both males (EAPC: −0.54%; 95% CI: −1.76% to 0.69%) and females (EAPC: −0.96%; 95% CI: −2.24% to 0.33%) (Figure 4B). The age-standardized stroke mortality increased in Pakistan whereas it decreased in all countries for females (Supplemental Tables 12 and 13).

During the projected period (2021-2040), the age-standardized stroke mortality rate in South Asia decreased (2021: 44.9 [95% CI: 44.7-45.0], 2040: 38.2 [95% CI: 27.8-48.5], EAPC: −0.85% [95% CI: −3.07% to 1.43%]) (Figure 3B) and this trend was observed in all the countries in South Asia (Table 1). The overall reduction in the age-standardized stroke mortality rate for South Asia is primarily driven by the projected decrease in the rates among females (EAPC: −1.54%; 95% CI: −3.94% to 0.91%) as the rate is projected to slightly increase among males (EAPC: 0.09%; 95% CI: −1.97% to 2.19%) (Figure 4B). In individual countries, stroke mortality rate in males is projected to decrease in every country except for Afghanistan and India, whereas for females, it is projected to decrease in each country (Supplemental Tables 12 and 13).

### PAD prevalence

The age-standardized PAD prevalence rate in South Asia increased between 1990 and 2021 (1990: 1,670.2 [95% CI: 1,668.5-1,671.8], 2021: 1,809.5 [95% CI: 1,808.5-1,810.6], EAPC: 0.26% [95% CI: 0.04%-0.47%]) (Figure 1C) and this trend was consistent across all nations (Supplemental Table 14). Males (EAPC: 0.26%; 95% CI: 0.01%-0.51%) were observed to have a higher rate of increase compared to females (EAPC: 0.17%; 95% CI: −0.02% to 0.36%) (Figure 2C). The age-standardized PAD prevalence rate increased for males and females in each South Asian country (Supplemental Tables 15 and 16). During the projected period (2021-2040), the age-standardized PAD prevalence rate in South Asia was expected to increase further (2021: 1,809.5 [95% CI: 1,808.5-1,810.6], 2040: 1,879.5 [95% CI: 1,684.9-2,074.0], EAPC: 0.26% [95% CI: 0.04%-0.47%]) (Figure 1C). This increasing trend was projected to occur in each country except the Maldives (EAPC: −0.24%; 95% CI: −0.51% to 0.04%) and Sri Lanka (EAPC: −0.01%; 95% CI: −0.27% to 0.26%) (Table 1). The overall increase in the PAD prevalence rate of South Asia was driven by the projected increase among females (EAPC: 0.29%; 95% CI: −0.01% to 0.59%) as the rate was expected to decrease slightly among males (EAPC: −0.06%; 95% CI: −0.46% to 0.34%) (Figure 2C). The PAD prevalence for males was projected to decrease in India and Bangladesh, whereas it was expected to increase in Afghanistan and Sri Lanka (Supplemental Table 15). However, the age-standardized PAD prevalence rate for females was projected to increase in all but 2 countries (the Maldives and Sri Lanka) (Supplemental Table 16).

## Discussion

This study projected the prevalence and mortality rates for ASCVD (specifically IHD, stroke, and PAD) in South Asia until 2040 using information from the GBD and population data from the Shared Socioeconomic Pathways. Overall, the age-adjusted IHD prevalence is projected to increase whereas mortality rates are likely to decrease. Age-adjusted stroke prevalence rates are likely to remain the same whereas mortality rates will decrease slightly and the age-adjusted PAD prevalence rates are expected to increase. More significantly, although the age-adjusted prevalence rates for all 3 conditions are expected to decrease among males, they will likely increase among females.

Globally, almost one-third of all non-accidental deaths can be attributed to CVD. The increasing prevalence of ASCVD is of particular relevance to the rapidly growing economies of LMIC, which also struggle with balancing growth with appropriate investments in health.^9^ Multiple prior studies have reported that the rates of traditional cardiovascular risk factors such as diabetes, obesity, and hypertension are have increased in the past decades are expected to continue an upward trajectory particularly in LMICs.^21^, ^22^, ^23^ The countries of South Asia which almost all belong to LMICs are also facing profound challenges due to worsening environmental and social issues partly attributable to expanding urbanization issues, all of which are expected to also increase in the future. Although traditional risk factors such as increasing diabetes and obesity are undoubtedly factors, environmental factors such as air pollution have been causally responsible for ASCVD and many of the risk factors such as hypertension and type 2 diabetes mellitus.^24^ A recent study reported that rapid urbanization, observed in many South Asian countries in the past decade, can result in rapid declines in green space availability.^25^ Adding these issues is the expected global warming which will result in more extreme weather events in South Asia in the coming decades.^26^^,^^27^ A 2020 air quality report stated that 37 of the top 40 polluted cities are in South Asia.^28^ Specifically, the small particulate matter air pollution concentration is extremely high in South Asian cities such as Delhi and Lahore.^29^

Using a Bayesian approach, we project increasing rates of ASCVD in this study. However, even more important is the sex inequity reported in our study. Our model projected that the prevalence of IHD and PAD will increase in females in most countries in South Asia. With better social mobility and economic independence, smoking rates among women in Asia are increasing.^30^ Furthermore, compared to males, females are less likely to receive appropriate medical therapy.^31^ A recent study from China reported that the women were less likely than men to receive primary and secondary CVD preventive therapy.^32^ Along with societal norms prevalent in South Asia that make it more challenging for females to receive timely care, studies have reported disparities in the treatments received by males and females. A recent study from India on patients presenting with acute coronary syndrome reported that females were less likely to receive guideline-directed medical therapy and percutaneous interventions despite having higher disease acuity.^33^ A study from South India also reported that females presenting with heart failure were less likely to receive guideline-directed medical therapy.^34^ Similar sex-related disparities in investigations and treatment were also reported by researchers in Pakistan. A large cohort study reported that, compared to males, females in Pakistan were observed to have higher prevalence of cardiovascular risk factors; additionally, another study reported that females were more likely to be non-adherent to lipid-lowering therapy in Pakistan.^35^^,^^36^ A large systematic review concluded that females are more likely to be receive appropriate medical therapy and increase physical activity; however, they are also more likely to make positive dietary changes if needed.^37^ The World Health Organization has introduced measures such as the Astana 2018 Declaration that attempts to improve investments in primary across 5 South Asian nations. Other nongovernmental and governmental organizations are working to reduce this sex disparity in health; however, many challenges still exist and our observations suggest that they may increase in the future.

Our study may have implications for the economic challenges already experienced by LMICs in South Asia. The lack of affordable universal health care policies, concentration of tertiary care facilities in bigger metropolitan cities, and poor public health policies has resulted in exclusion of a substantial majority of populations in this country from appropriate evidence-based cardiovascular therapies directed at traditional risk factors. A large cross-sectional study from communities across 18 LMICs reported that a large proportion of interviewed participants with CVD were unable to afford routine secondary preventive medications such as aspirin, beta-blockers, and statins.^38^ Unfortunately, a multicity, multinational cohort study from South Asia also reported that the use of inexpensive secondary preventive therapies such as statins, antiplatelet agents, and blood pressure–lowering drugs were suboptimal.^39^ Additionally, the lack of public policy instruments to regulate exposure to many toxic chemicals including air pollution and emerging climate threats are likely to amplify the cumulative adverse health impacts.

### Study limitations

Our study should be understood on the background of certain limitations. We have used data from the GBD, which itself uses a Bayesian model to obtain and harmonize estimates across nations. Therefore, results are dependent upon data processing from the GBD and prior probability assumptions which may be incorrect; additionally, future population estimates are projections considering country-level fertility, mortality, and migration rates. To provide the average estimates, we chose to use the SSP2 pathway. However, significant changes in either of these rates will also impact our observations. The present analysis does not take into account various health-related and environmental factors that can behave as confounders, moderators, or mediators of the ASCVD rates observed in our Bayesian models. The current mathematical modeling process does not account for these facts; hence, depending upon dynamic changes in these factors, the true prevalence of ASCVD in South Asia may vary from what we have observed in our study. In fact, given the trajectory at which the prevalence of CVD risk factors in South Asia are increasing, future IHD and PAD prevalence rates for these countries may, in fact, be higher than what we have reported. Many emerging health conditions, including environmental variables, involve complex interactions that are difficult to model with Bayesian inference, which may result in imprecise estimates. Finally in fast-changing medical scenarios, including climate and planetary risk factors, Bayesian models may take too long to adjust their predictions. But despite many of these constraints, we expect these observations to help with resource planning and appropriate shifts in policy to directly address the most important public health challenge for LMICs in South Asia.

## Conclusions

Our Bayesian modeling study demonstrated that ASCVD prevalence in South Asia is projected to increase while mortality may reduce in the coming decades. This increase in the overall rate may be driven by increases for IHD and PAD among females.

## Funding Support and Author Disclosures

The authors have reported that they have no relationships relevant to the contents of this paper to disclose.
