# Supplementary material for: Forecasting Atherosclerotic Cardiovascular Disease in South Asia Until 2040: A Bayesian Modeling Approach
Source: JACC Asia. 2025 Aug 21;5(10):1359–68. doi: 10.1016/j.jacasi.2025.06.014 (PMC12790086; doi:10.1016/j.jacasi.2025.06.014)
Supplement: Supplemental Material [file mmc1.docx]

**Forecasting Atherosclerotic cardiovascular disease in South Asia until 2040: A Bayesian modeling approach.**

**SUPPLEMENTAL MATERIAL**

Table of Contents

[Supplemental Table 1. Mid-year population estimates for South Asia. 3](#_Toc199224901)

[Supplemental Table 2: Age-Standardized IHD Prevalence Rates 4](#_Toc199224902)

[Supplemental Table 3. Male Age-Standardized IHD Prevalence Rates 5](#_Toc199224903)

[Supplemental Table 4. Female Age-Standardized IHD Prevalence Rates 6](#_Toc199224904)

[Supplemental Table 5: Age-Standardized IHD Mortality Rates 7](#_Toc199224905)

[Supplemental Table 6. Male Age-Standardized IHD Mortality Rates 8](#_Toc199224906)

[Supplemental Table 7. Female Age-Standardized IHD Mortality Rates 9](#_Toc199224907)

[Supplemental Table 8: Age-Standardized Stroke Prevalence Rates 10](#_Toc199224908)

[Supplemental Table 9. Male Age-Standardized Stroke Prevalence Rates 11](#_Toc199224909)

[Supplemental Table 10. Female Age-Standardized Stroke Prevalence Rates 12](#_Toc199224910)

[Supplemental Table 11: Age-Standardized Stroke Mortality Rates 13](#_Toc199224911)

[Supplemental Table 12. Male Age-Standardized Stroke Mortality Rates 14](#_Toc199224912)

[Supplemental Table 13. Female Age-Standardized Stroke Mortality Rates 15](#_Toc199224913)

[Supplemental Table 14: Age-Standardized PAD Prevalence Rates 16](#_Toc199224914)

[Supplemental Table 15. Male Age-Standardized PAD Prevalence Rates 17](#_Toc199224915)

[Supplemental Table 16. Female Age-Standardized PAD Prevalence Rates 18](#_Toc199224916)

[Supplemental Statistical Methods 19](#_Toc199224917)

# Supplemental Table 1. Mid-year population estimates for South Asia.

| **Country** | **Population 2021 (Ages 40-79)** | **Population 2040 (Ages 40-79)** | **Percent Change** |
| --- | --- | --- | --- |
| All South Asia | 543.2 | 850.4 | 56.5% |
| Afghanistan | 4.7 | 13.1 | 178.2% |
| Bangladesh | 48.1 | 78.9 | 64.1% |
| Bhutan | 0.2 | 0.4 | 87.3% |
| India | 423.2 | 649.8 | 53.5% |
| Maldives | 0.2 | 0.3 | 120.8% |
| Nepal | 8.2 | 11.9 | 44.9% |
| Pakistan | 50.0 | 85.9 | 72.0% |
| Sri Lanka | 8.7 | 10.0 | 15.2% |

This table presents the observed (2021) and projected (2040) mid-year population estimates (in millions) for South Asia and its constituent countries in the 40-79 year age bracket. The projected population is obtained from the Shared Socioeconomic Pathways Middle-of-the-road scenario.

# Supplemental Table 2: Age-Standardized IHD Prevalence Rates

| Year | All South Asia | Afghanistan | Bangladesh | Bhutan | India | Maldives | Nepal | Pakistan | Sri Lanka |
| --- | --- | --- | --- | --- | --- | --- | --- | --- | --- |
| 1990 | 8951.1 (8947.2, 8955.0) | 16983.3 (16931.4, 17035.3) | 8502.5 (8488.8, 8516.2) | 8430.9 (8248.5, 8616.2) | 8731.7 (8727.4, 8736.0) | 4060.0 (3852.6, 4275.7) | 8206.6 (8177.2, 8236.0) | 11382.9 (11368.3, 11397.6) | 4430.4 (4410.0, 4450.8) |
| 2000 | 9048.2 (9044.8, 9051.6) | 16475.1 (16425.0, 16525.2) | 8500.9 (8488.5, 8513.2) | 8510.9 (8354.5, 8669.5) | 8862.4 (8858.7, 8866.2) | 3854.6 (3686.5, 4028.3) | 8212.5 (8186.9, 8238.2) | 11664.7 (11651.0, 11678.5) | 4609.8 (4591.7, 4627.9) |
| 2010 | 9212.0 (9209.1, 9214.9) | 15629.0 (15578.7, 15679.4) | 8473.5 (8463.7, 8483.3) | 8632.2 (8494.2, 8771.8) | 9097.4 (9094.2, 9100.6) | 3694.5 (3554.9, 3837.9) | 8083.2 (8061.7, 8104.8) | 11883.2 (11870.9, 11895.5) | 4535.6 (4520.2, 4551.0) |
| 2021 | 9434.6 (9432.1, 9437.1) | 15531.6 (15483.6, 15579.8) | 8682.9 (8675.0, 8690.9) | 8768.8 (8648.3, 8890.5) | 9373.0 (9370.2, 9375.8) | 3806.6 (3699.2, 3916.4) | 7873.3 (7855.1, 7891.5) | 11823.5 (11813.3, 11833.7) | 4290.5 (4277.9, 4303.1) |
| 2030 | 9589.8 (9228.9, 9950.7) | 15928.9 (15223.4, 16634.4) | 8386.9 (8031.5, 8742.4) | 8974.3 (8501.3, 9447.2) | 9612.5 (9246.5, 9978.5) | 4065.1 (3790.3, 4340.0) | 7994.5 (7677.7, 8311.3) | 11520.1 (11044.2, 11996.0) | 4237.1 (4036.7, 4437.6) |
| 2040 | 9846.6 (8800.0, 10893.3) | 16781.1 (14712.3, 18849.9) | 8154.2 (7177.5, 9130.9) | 9269.5 (7949.5, 10589.5) | 9964.6 (8893.4, 11035.9) | 4480.7 (3688.5, 5273.0) | 8120.0 (7216.4, 9023.6) | 11259.8 (9953.8, 12565.8) | 4244.4 (3679.5, 4809.3) |
| Observed Results: Percent change (1990 – 2021) | | | | | | | | | |
|  | 5.4% | -8.5% | 2.1% | 4.0% | 7.3% | -6.2% | -4.1% | 3.9% | -3.2% |
| Observed Results: Estimated Annual Percent change (1990 – 2021) | | | | | | | | | |
|  | 0.17% (0.08%, 0.26%) | -0.29% (-0.36%, -0.22%) | 0.07% (-0.03%, 0.16%) | 0.13% (0.03%, 0.22%) | 0.23% (0.13%, 0.32%) | -0.21% (-0.35%, -0.07%) | -0.13% (-0.23%, -0.03%) | 0.12% (0.04%, 0.21%) | -0.1% (-0.24%, 0.03%) |
| Projected Results: Percent change (2021 – 2040) | | | | | | | | | |
|  | 4.4% | 8.0% | -6.1% | 5.7% | 6.3% | 17.7% | 3.1% | -4.8% | -1.1% |
| Projected Results: Estimated Annual percent change (2021 – 2040) | | | | | | | | | |
|  | 0.23% (0.08%, 0.37%) | 0.41% (0.29%, 0.52%) | -0.33% (-0.49%, -0.17%) | 0.29% (0.14%, 0.45%) | 0.32% (0.17%, 0.47%) | 0.86% (0.63%, 1.09%) | 0.16% (0%, 0.33%) | -0.26% (-0.39%, -0.12%) | -0.06% (-0.28%, 0.17%) |

This table reports the age-standardized prevalence rates (per 100,000 individuals) of ischemic heart disease (IHD) in South Asia between 1990 and 2040. The age-standardized rates for the observed years (1990-2021) are reported with 95% confidence intervals. The age-standardized rates calculated using the Bayesian Age-Period-Cohort (BAPC) projections (2030, 2040) are reported as median value (25^th^ percentile, 75^th^ percentile). To evaluate the temporal change for each period we calculated the percent change between the initial and final value for that period. We further reported the estimated annual percentage change over the period. Confidence intervals for the estimated annual percentage change are calculated using bootstrap.

# Supplemental Table 3. Male Age-Standardized IHD Prevalence Rates

| Year | All South Asia | Afghanistan | Bangladesh | Bhutan | India | Maldives | Nepal | Pakistan | Sri Lanka |
| --- | --- | --- | --- | --- | --- | --- | --- | --- | --- |
| 1990 | 10721.7 (10715.9, 10727.5) | 22443.7 (22359.1, 22528.6) | 10542.4 (10521.7, 10563.1) | 10242.7 (9964.5, 10526.7) | 10285.2 (10278.8, 10291.6) | 5305.4 (4987.4, 5638.4) | 10078.2 (10032.8, 10123.7) | 14475.4 (14452.7, 14498.1) | 5846.2 (5813.1, 5879.4) |
| 2000 | 11053.4 (11048.2, 11058.6) | 22210.9 (22125.9, 22296.2) | 10641.8 (10623.1, 10660.4) | 10260.8 (10025.4, 10500.4) | 10669.5 (10663.8, 10675.3) | 5147.4 (4879.4, 5426) | 10256.2 (10216, 10296.6) | 15049.9 (15028.2, 15071.6) | 6365.7 (6334.9, 6396.7) |
| 2010 | 11437.7 (11433.1, 11442.3) | 21621.5 (21530.1, 21713.2) | 10747.8 (10732.6, 10763) | 10439.4 (10230.3, 10651.6) | 11170.5 (11165.4, 11175.6) | 5005.1 (4775, 5242.9) | 10180.8 (10146.4, 10215.2) | 15277.1 (15257.5, 15296.8) | 6213.8 (6187.2, 6240.6) |
| 2021 | 11425.6 (11421.7, 11429.5) | 21463.3 (21372, 21554.9) | 11011.5 (10998.9, 11024.2) | 10582.9 (10398.6, 10769.6) | 11140.3 (11136, 11144.6) | 5050.5 (4877.2, 5228.2) | 10059.1 (10029.1, 10089.3) | 15265.4 (15248.9, 15282) | 5762.9 (5741.3, 5784.6) |
| 2030 | 11126.8 (10679.5, 11574.1) | 21113.5 (20137.3, 22089.6) | 10432.7 (9947, 10918.4) | 10738.1 (10136.6, 11339.5) | 10851 (10409.5, 11292.5) | 5177.9 (4806.5, 5549.4) | 10198.5 (9779.3, 10617.7) | 14817.8 (14183.9, 15451.6) | 5490 (5184.9, 5795.1) |
| 2040 | 10898.1 (9664.9, 12131.2) | 20945.2 (18262.3, 23628.1) | 9886.6 (8587.4, 11185.8) | 10962 (9317.1, 12606.8) | 10628.1 (9412.2, 11844) | 5361.4 (4368.3, 6354.5) | 10336.2 (9144.5, 11527.9) | 14474.9 (12735.4, 16214.3) | 5299.5 (4470, 6129) |
| Observed Results: Percent change (1990 – 2021) | | | | | | | | | |
|  | 6.6% | -4.4% | 4.5% | 3.3% | 8.3% | -4.8% | -0.2% | 5.5% | -1.4% |
| Observed Results: Estimated Annual Percent change (1990 – 2021) | | | | | | | | | |
|  | 0.21% (0.12%, 0.29%) | -0.14% (-0.20%, -0.08%) | 0.14% (0.05%, 0.23%) | 0.11% (0.02%, 0.19%) | 0.26% (0.17%, 0.34%) | -0.16% (-0.28%, -0.03%) | -0.01% (-0.10%, 0.08%) | 0.17% (0.10%, 0.25%) | -0.05% (-0.16%, 0.07%) |
| Projected Results: Percent change (2021 – 2040) | | | | | | | | | |
|  | -4.6% | -2.4% | -10.2% | 3.6% | -4.6% | 6.2% | 2.8% | -5.2% | -8% |
| Projected Results: Estimated Annual percent change (2021 – 2040) | | | | | | | | | |
|  | -0.25% (-0.39%, -0.11%) | -0.13% (-0.23%, -0.03%) | -0.57% (-0.71%, -0.42%) | 0.19% (0.04%, 0.33%) | -0.25% (-0.39%, -0.11%) | 0.31% (0.11%, 0.52%) | 0.14% (0%, 0.29%) | -0.28% (-0.4%, -0.16%) | -0.44% (-0.64%, -0.24%) |

This table reports the age-standardized prevalence rates (per 100,000 individuals) of ischemic heart disease (IHD) among males in South Asia between 1990 and 2040. The age-standardized rates for the observed years (1990-2021) are reported with 95% confidence intervals. The age-standardized rates calculated using the Bayesian Age-Period-Cohort (BAPC) projections (2030, 2040) are reported as median value (25^th^ percentile, 75^th^ percentile). To evaluate the temporal change for each period we calculated the percent change between the initial and final value for that period. We further reported the estimated annual percentage change over the period. Confidence intervals for the estimated annual percentage change are calculated using bootstrap.

# Supplemental Table 4. Female Age-Standardized IHD Prevalence Rates

| Year | All South Asia | Afghanistan | Bangladesh | Bhutan | India | Maldives | Nepal | Pakistan | Sri Lanka |
| --- | --- | --- | --- | --- | --- | --- | --- | --- | --- |
| 1990 | 6977.7 (6972.7, 6982.6) | 11581.2 (11520.8, 11641.9) | 6057.4 (6040.3, 6074.6) | 6470.4 (6240.7, 6706.5) | 7006.3 (7000.8, 7011.9) | 2510.1 (2269, 2770) | 6234 (6197.4, 6270.8) | 7887.3 (7869.5, 7905.1) | 3002.6 (2978.8, 3026.5) |
| 2000 | 6935.3 (6931, 6939.5) | 11383.6 (11326.6, 11440.7) | 5890.6 (5875.3, 5905.9) | 6466.9 (6268.4, 6670.2) | 6971.9 (6967.1, 6976.6) | 2407.1 (2216.1, 2609.8) | 6099.7 (6068.2, 6131.2) | 8027.8 (8011.5, 8044) | 3021.2 (3001.1, 3041.5) |
| 2010 | 6977.2 (6973.6, 6980.8) | 10996.5 (10941.1, 11052) | 5901.6 (5889.6, 5913.5) | 6540.7 (6366.8, 6718.1) | 7028.8 (7024.8, 7032.8) | 2346.8 (2191.8, 2509.3) | 6009.2 (5983.1, 6035.4) | 8332.8 (8318.3, 8347.4) | 3132.8 (3115.5, 3150.1) |
| 2021 | 7478.6 (7475.5, 7481.8) | 11160.9 (11109.2, 11212.8) | 6304.7 (6295.1, 6314.3) | 6765.1 (6613.3, 6919.4) | 7635.9 (7632.4, 7639.5) | 2406.5 (2283.9, 2533.9) | 5935.8 (5914.2, 5957.4) | 8306.3 (8294.2, 8318.3) | 3073.8 (3059.5, 3088.1) |
| 2030 | 8276.5 (7941.4, 8611.7) | 11845.6 (11316.9, 12374.3) | 6596.6 (6326.8, 6866.5) | 7031.3 (6601, 7461.6) | 8608.5 (8242.8, 8974.3) | 2476 (2269.7, 2682.2) | 6277.1 (5992.5, 6561.7) | 8290 (7937, 8643) | 3172.1 (3013.9, 3330.3) |
| 2040 | 9305.5 (8240.6, 10370.3) | 12892.2 (11292.5, 14491.9) | 7005 (6195.8, 7814.2) | 7396.8 (6202.2, 8591.4) | 9867.4 (8684.7, 11050.1) | 2591.6 (2046.6, 3136.6) | 6705 (5855.8, 7554.3) | 8310.7 (7312.7, 9308.7) | 3298.4 (2841.2, 3755.7) |
| Observed Results: Percent change (1990 – 2021) | | | | | | | | | |
|  | 7.2% | -3.6% | 4.1% | 4.6% | 9% | -4.1% | -4.8% | 5.3% | 2.4% |
| Observed Results: Estimated Annual Percent change (1990 – 2021) | | | | | | | | | |
|  | 0.22% (0.12%, 0.33%) | -0.12% (-0.2%, -0.04%) | 0.13% (0.02%, 0.24%) | 0.14% (0.03%, 0.25%) | 0.28% (0.17%, 0.38%) | -0.14% (-0.32%, 0.04%) | -0.16% (-0.27%, -0.04%) | 0.17% (0.07%, 0.27%) | 0.08% (-0.09%, 0.24%) |
| Projected Results: Percent change (2021 – 2040) | | | | | | | | | |
|  | 24.4% | 15.5% | 11.1% | 9.3% | 29.2% | 7.7% | 13% | 0.1% | 7.3% |
| Projected Results: Estimated Annual percent change (2021 – 2040) | | | | | | | | | |
|  | 1.16% (1%, 1.32%) | 0.76% (0.63%, 0.9%) | 0.56% (0.38%, 0.74%) | 0.47% (0.3%, 0.65%) | 1.36% (1.2%, 1.52%) | 0.39% (0.1%, 0.68%) | 0.64% (0.46%, 0.83%) | 0% (-0.16%, 0.16%) | 0.37% (0.11%, 0.63%) |

This table reports the age-standardized prevalence rates (per 100,000 individuals) of ischemic heart disease (IHD) among females in South Asia between 1990 and 2040. The age-standardized rates for the observed years (1990-2021) are reported with 95% confidence intervals. The age-standardized rates calculated using the Bayesian Age-Period-Cohort (BAPC) projections (2030, 2040) are reported as median value (25^th^ percentile, 75^th^ percentile). To evaluate the temporal change for each period we calculated the percent change between the initial and final value for that period. We further reported the estimated annual percentage change over the period. Confidence intervals for the estimated annual percentage change are calculated using bootstrap.

# Supplemental Table 5: Age-Standardized IHD Mortality Rates

| Year | All South Asia | Afghanistan | Bangladesh | Bhutan | India | Maldives | Nepal | Pakistan | Sri Lanka |
| --- | --- | --- | --- | --- | --- | --- | --- | --- | --- |
| 1990 | 261.0 (260.4, 261.7) | 779.7 (768.7, 791.0) | 247.5 (245.2, 249.9) | 188.8 (162.4, 218.3) | 256.6 (255.9, 257.3) | 280.3 (227.8, 341.3) | 234.6 (229.7, 239.6) | 264.4 (262.1, 266.6) | 217.6 (213.1, 222.2) |
| 2000 | 254.7 (254.2, 255.3) | 774.1 (763.2, 785.0) | 204.3 (202.4, 206.2) | 176.4 (154.5, 200.4) | 248.6 (248.0, 249.2) | 203.9 (166.8, 246.6) | 204.7 (200.6, 208.7) | 323.3 (321.0, 325.6) | 220.6 (216.6, 224.6) |
| 2010 | 253.1 (252.6, 253.6) | 615.3 (605.5, 625.2) | 196.8 (195.3, 198.3) | 166.3 (147.8, 186.4) | 251.1 (250.6, 251.6) | 121.2 (97.5, 148.7) | 215.0 (211.5, 218.5) | 325.3 (323.3, 327.4) | 191.3 (188.2, 194.5) |
| 2021 | 254.7 (254.3, 255.1) | 549.8 (540.9, 558.8) | 179.3 (178.2, 180.5) | 159.3 (143.6, 176.2) | 257.2 (256.7, 257.7) | 88.2 (72.6, 106.2) | 211.3 (208.3, 214.3) | 329.1 (327.4, 330.8) | 146.8 (144.5, 149.2) |
| 2030 | 239.7 (217.6, 261.9) | 520.8 (483.2, 558.5) | 161.3 (146.8, 175.7) | 148.5 (132.7, 164.3) | 245.1 (218.9, 271.4) | 65.4 (55.1, 75.7) | 202.7 (182.2, 223.3) | 307.0 (278.1, 335.9) | 133.2 (93.8, 172.6) |
| 2040 | 224.0 (166.5, 281.6) | 515.1 (412.6, 617.5) | 142.7 (108.1, 177.3) | 133.7 (99.7, 167.7) | 230.9 (162.4, 299.3) | 50.3 (31.9, 68.7) | 189.4 (135.6, 243.2) | 286.7 (209.9, 363.6) | 123.6 (19.4, 227.8) |
| Observed Results: Percent change (1990 – 2021) | | | | | | | | | |
|  | -2.4% | -29.5% | -27.5% | -15.6% | 0.2% | -68.5% | -9.9% | 24.5% | -32.5% |
| Observed Results: Estimated Annual Percent change (1990 – 2021) | | | | | | | | | |
|  | -0.08% (-0.63%, 0.48%) | -1.12% (-1.47%, -0.77%) | -1.03% (-1.65%, -0.42%) | -0.55% (-1.22%, 0.13%) | 0.01% (-0.55%, 0.57%) | -3.66% (-4.4%, -2.91%) | -0.34% (-0.93%, 0.26%) | 0.71% (0.18%, 1.24%) | -1.26% (-1.93%, -0.59%) |
| Projected Results: Percent change (2021 – 2040) | | | | | | | | | |
|  | -12.0% | -6.3% | -20.4% | -16.1% | -10.2% | -43.0% | -10.3% | -12.9% | -15.8% |
| Projected Results: Estimated Annual percent change (2021 – 2040) | | | | | | | | | |
|  | -0.67% (-1.61%, 0.27%) | -0.34% (-0.97%, 0.29%) | -1.19% (-2.33%, -0.04%) | -0.92% (-2.11%, 0.29%) | -0.57% (-1.49%, 0.37%) | -2.91% (-4.67%, -1.13%) | -0.57% (-1.6%, 0.46%) | -0.72% (-1.55%, 0.11%) | -0.9% (-2.14%, 0.35%) |

This table reports the age-standardized mortality rates (per 100,000 individuals) of ischemic heart disease (IHD) in South Asia between 1990 and 2040. The age-standardized rates for the observed years (1990-2021) are reported with 95% confidence intervals. The age-standardized rates calculated using the Bayesian Age-Period-Cohort (BAPC) projections (2030, 2040) are reported as median value (25^th^ percentile, 75^th^ percentile). To evaluate the temporal change for each period we calculated the percent change between the initial and final value for that period. We further reported the estimated annual percentage change over the period. Confidence intervals for the estimated annual percentage change are calculated using bootstrap.

# Supplemental Table 6. Male Age-Standardized IHD Mortality Rates

| Year | All South Asia | Afghanistan | Bangladesh | Bhutan | India | Maldives | Nepal | Pakistan | Sri Lanka |
| --- | --- | --- | --- | --- | --- | --- | --- | --- | --- |
| 1990 | 307.8 (306.9, 308.8) | 984.5 (966.8, 1002.4) | 307.1 (303.6, 310.7) | 209.4 (171.3, 253.5) | 306 (304.9, 307.1) | 317.2 (243.2, 406.6) | 249.1 (242, 256.4) | 264 (261, 267.1) | 287.5 (280.2, 294.9) |
| 2000 | 314.7 (313.8, 315.6) | 982.8 (964.9, 1000.9) | 263 (260, 265.9) | 199.2 (167.6, 235) | 311.4 (310.4, 312.4) | 244.5 (188.8, 311.1) | 236.8 (230.7, 243) | 340.2 (337, 343.5) | 313.1 (306.3, 320) |
| 2010 | 327.8 (327.1, 328.6) | 779.8 (762.7, 797.2) | 256.5 (254.1, 258.8) | 203.9 (175.7, 235.1) | 329.5 (328.7, 330.4) | 165.4 (126.3, 212.1) | 266.9 (261.4, 272.5) | 368.3 (365.3, 371.4) | 277.5 (271.9, 283.2) |
| 2021 | 325.8 (325.1, 326.4) | 690.8 (674.8, 707) | 230.2 (228.3, 232) | 199.5 (175.2, 226.2) | 332.1 (331.3, 332.8) | 118.9 (93.6, 148.7) | 286.9 (281.9, 292) | 370.9 (368.3, 373.5) | 209.7 (205.6, 213.9) |
| 2030 | 326.4 (294.8, 358.1) | 647 (598.7, 695.3) | 217.6 (194.2, 240.9) | 186.9 (165.1, 208.8) | 339.4 (300.7, 378.1) | 83.5 (70.8, 96.2) | 281.9 (250.8, 313) | 350 (320.1, 379.9) | 193.5 (124.4, 262.6) |
| 2040 | 327.3 (239.6, 414.9) | 632.2 (504.4, 760.1) | 201.2 (142.9, 259.4) | 167.6 (121.4, 213.8) | 346.3 (237.5, 455) | 60.1 (39.7, 80.6) | 270.4 (187.3, 353.4) | 331.9 (251.8, 412) | 183.6 (-4.2, 371.4) |
| Observed Results: Percent change (1990 – 2021) | | | | | | | | | |
|  | 5.8% | -29.8% | -25% | -4.7% | 8.5% | -62.5% | 15.2% | 40.5% | -27% |
| Observed Results: Estimated Annual Percent change (1990 – 2021) | | | | | | | | | |
|  | 0.18% (-0.32%, 0.69%) | -1.14% (-1.45%, -0.83%) | -0.93% (-1.47%, -0.38%) | -0.16% (-0.78%, 0.47%) | 0.26% (-0.24%, 0.77%) | -3.12% (-3.77%, -2.45%) | 0.46% (-0.09%, 1.01%) | 1.1% (0.59%, 1.62%) | -1.01% (-1.58%, -0.44%) |
| Projected Results: Percent change (2021 – 2040) | | | | | | | | | |
|  | 0.5% | -8.5% | -12.6% | -16% | 4.3% | -49.4% | -5.8% | -10.5% | -12.5% |
| Projected Results: Estimated Annual percent change (2021 – 2040) | | | | | | | | | |
|  | 0.02% (-0.78%, 0.83%) | -0.47% (-1.03%, 0.1%) | -0.71% (-1.69%, 0.29%) | -0.91% (-1.98%, 0.16%) | 0.22% (-0.57%, 1.02%) | -3.53% (-5.09%, -1.94%) | -0.31% (-1.18%, 0.56%) | -0.58% (-1.35%, 0.19%) | -0.7% (-1.73%, 0.34%) |

This table reports the age-standardized mortality rates (per 100,000 individuals) of ischemic heart disease (IHD) among males in South Asia between 1990 and 2040. The age-standardized rates for the observed years (1990-2021) are reported with 95% confidence intervals. The age-standardized rates calculated using the Bayesian Age-Period-Cohort (BAPC) projections (2030, 2040) are reported as median value (25^th^ percentile, 75^th^ percentile). To evaluate the temporal change for each period we calculated the percent change between the initial and final value for that period. We further reported the estimated annual percentage change over the period. Confidence intervals for the estimated annual percentage change are calculated using bootstrap.

# Supplemental Table 7. Female Age-Standardized IHD Mortality Rates

| Year | All South Asia | Afghanistan | Bangladesh | Bhutan | India | Maldives | Nepal | Pakistan | Sri Lanka |
| --- | --- | --- | --- | --- | --- | --- | --- | --- | --- |
| 1990 | 208.9 (208, 209.8) | 577.1 (563.7, 590.8) | 176.1 (173.2, 179.1) | 164.4 (129.5, 205.7) | 201.7 (200.8, 202.7) | 253.5 (181.1, 345.3) | 219.2 (212.4, 226.2) | 264.8 (261.5, 268) | 147.2 (142, 152.6) |
| 2000 | 191.1 (190.4, 191.8) | 590 (577.1, 603.1) | 132.1 (129.8, 134.5) | 148.9 (120.2, 182.4) | 182.4 (181.6, 183.2) | 157.6 (112.5, 214.4) | 171.4 (166.2, 176.7) | 302.9 (299.8, 306.1) | 135.9 (131.6, 140.2) |
| 2010 | 177.3 (176.8, 177.9) | 485.4 (473.9, 497) | 128.5 (126.7, 130.2) | 125.5 (102.7, 151.7) | 172.2 (171.6, 172.8) | 73.5 (48.9, 105.2) | 163.6 (159.3, 167.9) | 276.7 (274, 279.4) | 118.3 (115, 121.7) |
| 2021 | 184.6 (184.1, 185) | 438.6 (428.5, 448.9) | 128.4 (127.1, 129.8) | 114.5 (95.8, 135.7) | 183.1 (182.5, 183.6) | 52 (35.7, 73.1) | 144.5 (141.1, 147.8) | 282.9 (280.6, 285.1) | 93.8 (91.4, 96.3) |
| 2030 | 158.9 (139.9, 178) | 408 (375.6, 440.5) | 112.9 (99.4, 126.4) | 103.3 (89, 117.6) | 156.6 (135.3, 177.9) | 33.5 (26.1, 41) | 137 (121.1, 153) | 262 (230.5, 293.4) | 75.5 (62, 89) |
| 2040 | 133.3 (89.3, 177.4) | 386.1 (303.3, 469) | 98.6 (66.6, 130.6) | 92 (62.6, 121.4) | 128.5 (80.7, 176.3) | 22 (11.3, 32.7) | 129.4 (88, 170.7) | 242.1 (158.9, 325.3) | 61.5 (30.9, 92.1) |
| Observed Results: Percent change (1990 – 2021) | | | | | | | | | |
|  | -11.7% | -24% | -27.1% | -30.3% | -9.3% | -79.5% | -34.1% | 6.8% | -36.3% |
| Observed Results: Estimated Annual Percent change (1990 – 2021) | | | | | | | | | |
|  | -0.4% (-1.03%, 0.24%) | -0.88% (-1.28%, -0.48%) | -1.01% (-1.74%, -0.28%) | -1.16% (-1.92%, -0.4%) | -0.31% (-0.95%, 0.33%) | -4.98% (-5.89%, -4.06%) | -1.34% (-2%, -0.66%) | 0.21% (-0.33%, 0.76%) | -1.44% (-2.26%, -0.62%) |
| Projected Results: Percent change (2021 – 2040) | | | | | | | | | |
|  | -27.7% | -12% | -23.2% | -19.7% | -29.8% | -57.7% | -10.5% | -14.4% | -34.4% |
| Projected Results: Estimated Annual percent change (2021 – 2040) | | | | | | | | | |
|  | -1.7% (-2.84%, -0.54%) | -0.67% (-1.38%, 0.05%) | -1.38% (-2.73%, -0.01%) | -1.14% (-2.56%, 0.29%) | -1.85% (-3%, -0.67%) | -4.43% (-6.9%, -1.89%) | -0.58% (-1.81%, 0.67%) | -0.82% (-1.71%, 0.08%) | -2.2% (-3.84%, -0.53%) |

This table reports the age-standardized mortality rates (per 100,000 individuals) of ischemic heart disease (IHD) among females in South Asia between 1990 and 2040. The age-standardized rates for the observed years (1990-2021) are reported with 95% confidence intervals. The age-standardized rates calculated using the Bayesian Age-Period-Cohort (BAPC) projections (2030, 2040) are reported as median value (25^th^ percentile, 75^th^ percentile). To evaluate the temporal change for each period we calculated the percent change between the initial and final value for that period. We further reported the estimated annual percentage change over the period. Confidence intervals for the estimated annual percentage change are calculated using bootstrap.

# Supplemental Table 8: Age-Standardized Stroke Prevalence Rates

| Year | All South Asia | Afghanistan | Bangladesh | Bhutan | India | Maldives | Nepal | Pakistan | Sri Lanka |
| --- | --- | --- | --- | --- | --- | --- | --- | --- | --- |
| 1990 | 1142.3 (1140.9, 1143.7) | 2056.8 (2038.8, 2075.0) | 1350.1 (1344.6, 1355.6) | 1154.8 (1087.9, 1224.6) | 1037.2 (1035.8, 1038.7) | 2347.1 (2190.0, 2512.5) | 1073.9 (1063.3, 1084.6) | 1674.1 (1668.5, 1679.7) | 1780.5 (1767.6, 1793.5) |
| 2000 | 1121.2 (1120.0, 1122.4) | 2040.1 (2022.1, 2058.3) | 1287.8 (1283.0, 1292.6) | 1126.6 (1069.6, 1185.9) | 1027.0 (1025.7, 1028.3) | 2061.4 (1936.1, 2192.4) | 1033.5 (1024.4, 1042.7) | 1681.5 (1676.3, 1686.7) | 1760.0 (1744.8, 1767.2) |
| 2010 | 1072.2 (1071.2, 1073.2) | 2042.4 (2023.9, 2061.0) | 1253.4 (1249.6, 1257.2) | 1084.4 (1035.1, 1135.4) | 975.3 (974.2, 976.4) | 1592.0 (1499.8, 1688.1) | 974.9 (967.3, 982.5) | 1686.7 (1682.1, 1691.3) | 1652.3 (1643.0, 1661.7) |
| 2021 | 1065.5 (1064.7, 1066.4) | 2010.1 (1993.3, 2026.9) | 1289.0 (1285.9, 1292.1) | 1071.8 (1029.4, 1115.6) | 960.9 (960.0, 961.8) | 1382.6 (1318.7, 1448.7) | 951.1 (944.7, 957.5) | 1663.1 (1659.3, 1666.9) | 1635.9 (1627.8, 1644.1) |
| 2030 | 1067.7 (1025.4, 1110.0) | 1982.8 (1892.5, 2073.0) | 1325.3 (1265.8, 1384.8) | 1066.6 (988.3, 1144.9) | 956.7 (917.0, 996.3) | 1285.4 (1174.9, 1395.8) | 940.8 (898.8, 982.7) | 1643.6 (1580.3, 1707.0) | 1561.7 (1450.4, 1673.1) |
| 2040 | 1074.6 (953.7, 1195.5) | 1924.7 (1682.6, 2166.7) | 1388.4 (1211.3, 1565.5) | 1069.0 (866.7, 1271.2) | 957.5 (844.7, 1070.3) | 1197.1 (932.8, 1461.4) | 932.9 (817.1, 1048.8) | 1635.8 (1457.3, 1814.2) | 1428.9 (1160.4, 1697.4) |
| Observed Results: Percent change (1990 – 2021) | | | | | | | | | |
|  | -6.7% | -2.3% | -4.5% | -7.2% | -7.4% | -41.1% | -11.4% | -0.7% | -8.1% |
| Observed Results: Estimated Annual Percent change (1990 – 2021) | | | | | | | | | |
|  | -0.22% (-0.49%, 0.04%) | -0.07% (-0.27%, 0.12%) | -0.15% (-0.39%, 0.1%) | -0.24% (-0.51%, 0.03%) | -0.25% (-0.53%, 0.04%) | -1.69% (-1.9%, -1.48%) | -0.39% (-0.67%, -0.11%) | -0.02% (-0.24%, 0.2%) | -0.27% (-0.49%, -0.06%) |
| Projected Results: Percent change (2021 – 2040) | | | | | | | | | |
|  | 0.8% | -4.2% | 7.7% | -0.3% | -0.3% | -13.4% | -1.9% | -1.6% | -12.7% |
| Projected Results: Estimated Annual percent change (2021 – 2040) | | | | | | | | | |
|  | 0.04% (-0.4%, 0.49%) | -0.23% (-0.56%, 0.1%) | 0.39% (-0.01%, 0.79%) | -0.01% (-0.46%, 0.43%) | -0.02% (-0.49%, 0.45%) | -0.76% (-1.16%, -0.35%) | -0.1% (-0.58%, 0.37%) | -0.09% (-0.45%, 0.27%) | -0.71% (-1.08%, -0.34%) |

This table reports the age-standardized prevalence rates (per 100,000 individuals) of stroke in South Asia between 1990 and 2040. The age-standardized rates for the observed years (1990-2021) are reported with 95% confidence intervals. The age-standardized rates calculated using the Bayesian Age-Period-Cohort (BAPC) projections (2030, 2040) are reported as median value (25^th^ percentile, 75^th^ percentile). To evaluate the temporal change for each period we calculated the percent change between the initial and final value for that period. We further reported the estimated annual percentage change over the period. Confidence intervals for the estimated annual percentage change are calculated using bootstrap.

# Supplemental Table 9. Male Age-Standardized Stroke Prevalence Rates

| Year | All South Asia | Afghanistan | Bangladesh | Bhutan | India | Maldives | Nepal | Pakistan | Sri Lanka |
| --- | --- | --- | --- | --- | --- | --- | --- | --- | --- |
| 1990 | 1270 (1268, 1272) | 2378.2 (2350.7, 2405.9) | 1563.9 (1555.9, 1571.8) | 1220.5 (1125.7, 1321.2) | 1153.4 (1151.3, 1155.6) | 2522.3 (2304.5, 2755.1) | 1180.3 (1164.8, 1195.9) | 1811.2 (1803.2, 1819.2) | 1906.5 (1887.6, 1925.5) |
| 2000 | 1267.2 (1265.4, 1269) | 2373.2 (2344.9, 2401.8) | 1488.8 (1481.9, 1495.8) | 1195 (1114.8, 1279.3) | 1163.4 (1161.4, 1165.3) | 2260.6 (2081.2, 2451.1) | 1146.8 (1133.3, 1160.4) | 1823.8 (1816.3, 1831.3) | 1902.1 (1885.3, 1919) |
| 2010 | 1222.2 (1220.6, 1223.7) | 2420.1 (2389.2, 2451.2) | 1449.2 (1443.6, 1454.8) | 1154.2 (1084.6, 1227) | 1118.1 (1116.4, 1119.7) | 1792.4 (1655.1, 1937.5) | 1094.1 (1082.8, 1105.5) | 1813.3 (1806.6, 1820) | 1766.9 (1752.7, 1781.1) |
| 2021 | 1194.9 (1193.7, 1196.2) | 2413.7 (2384, 2443.7) | 1524 (1519.2, 1528.9) | 1141.4 (1080.8, 1204.4) | 1078.8 (1077.4, 1080.2) | 1555.3 (1461.3, 1653.6) | 1076 (1066, 1086) | 1772.6 (1767, 1778.1) | 1710.7 (1698.5, 1722.9) |
| 2030 | 1162.4 (1116.8, 1208.1) | 2410.4 (2291.9, 2528.9) | 1523.6 (1384.2, 1662.9) | 1132.8 (1040.9, 1224.7) | 1046.3 (1003.8, 1088.9) | 1429.7 (1298.7, 1560.8) | 1061.3 (1010.7, 1111.8) | 1712.6 (1644.7, 1780.6) | 1615.3 (1514.6, 1716.1) |
| 2040 | 1130.4 (1004.5, 1256.4) | 2388.9 (2068.3, 2709.5) | 1545.2 (1145.3, 1945.1) | 1131.3 (899.3, 1363.2) | 1014.9 (898.5, 1131.3) | 1308.7 (1007.5, 1609.8) | 1041.7 (905.3, 1178.2) | 1664.1 (1478.3, 1849.9) | 1435.3 (1190.5, 1680.1) |
| Observed Results: Percent change (1990 – 2021) | | | | | | | | | |
|  | -5.9% | 1.5% | -2.5% | -6.5% | -6.5% | -38.3% | -8.8% | -2.1% | -10.3% |
| Observed Results: Estimated Annual Percent change (1990 – 2021) | | | | | | | | | |
|  | -0.2% (-0.45%, 0.06%) | 0.05% (-0.13%, 0.23%) | -0.08% (-0.31%, 0.14%) | -0.22% (-0.48%, 0.04%) | -0.22% (-0.48%, 0.05%) | -1.55% (-1.75%, -1.35%) | -0.30% (-0.56%, -0.03%) | -0.07% (-0.28%, 0.14%) | -0.35% (-0.56%, -0.14%) |
| Projected Results: Percent change (2021 – 2040) | | | | | | | | | |
|  | -5.4% | -1% | 1.4% | -0.9% | -5.9% | -15.9% | -3.2% | -6.1% | -16.1% |
| Projected Results: Estimated Annual percent change (2021 – 2040) | | | | | | | | | |
|  | -0.29% (-0.72%, 0.14%) | -0.05% (-0.35%, 0.24%) | 0.07% (-0.3%, 0.45%) | -0.05% (-0.48%, 0.39%) | -0.32% (-0.77%, 0.13%) | -0.9% (-1.29%, -0.52%) | -0.17% (-0.62%, 0.28%) | -0.33% (-0.68%, 0.02%) | -0.92% (-1.28%, -0.55%) |

This table reports the age-standardized prevalence rates (per 100,000 individuals) of stroke among males in South Asia between 1990 and 2040. The age-standardized rates for the observed years (1990-2021) are reported with 95% confidence intervals. The age-standardized rates calculated using the Bayesian Age-Period-Cohort (BAPC) projections (2030, 2040) are reported as median value (25^th^ percentile, 75^th^ percentile). To evaluate the temporal change for each period we calculated the percent change between the initial and final value for that period. We further reported the estimated annual percentage change over the period. Confidence intervals for the estimated annual percentage change are calculated using bootstrap.

# Supplemental Table 10. Female Age-Standardized Stroke Prevalence Rates

| Year | All South Asia | Afghanistan | Bangladesh | Bhutan | India | Maldives | Nepal | Pakistan | Sri Lanka |
| --- | --- | --- | --- | --- | --- | --- | --- | --- | --- |
| 1990 | 1000 (998.1, 1001.9) | 1738.9 (1715.5, 1762.5) | 1093.8 (1086.5, 1101.1) | 1083.5 (990.6, 1182.6) | 908.2 (906.2, 910.2) | 2123.5 (1902.1, 2363.5) | 961.8 (947.4, 976.3) | 1519.1 (1511.3, 1526.9) | 1653.5 (1635.8, 1671.2) |
| 2000 | 969.9 (968.3, 971.5) | 1735.9 (1713.1, 1759) | 1036.2 (1029.8, 1042.6) | 1047.2 (967.2, 1132.1) | 885.3 (883.6, 887) | 1828.7 (1657.8, 2011.9) | 916.6 (904.4, 929) | 1530.1 (1523.1, 1537.2) | 1615.4 (1600.7, 1630.3) |
| 2010 | 924 (922.6, 925.3) | 1741.3 (1718.8, 1763.9) | 1032.2 (1027.2, 1037.2) | 1002.9 (934.2, 1075.4) | 833.8 (832.4, 835.3) | 1389.3 (1268, 1518.6) | 856.9 (847, 867) | 1557.4 (1551.2, 1563.6) | 1529.4 (1517.2, 1541.7) |
| 2021 | 942.1 (941, 943.2) | 1716.9 (1697.4, 1736.6) | 1093.4 (1089.3, 1097.4) | 996.2 (937.3, 1057.7) | 845.3 (844.1, 846.6) | 1194.5 (1107.7, 1286.2) | 839 (830.7, 847.4) | 1550 (1544.9, 1555.2) | 1482.9 (1472.6, 1493.4) |
| 2030 | 981.3 (939.3, 1023.4) | 1674.2 (1591.9, 1756.5) | 1176 (1122.3, 1229.7) | 994.7 (909.5, 1080) | 871.8 (831.3, 912.4) | 1099.1 (988.2, 1209.9) | 837.9 (796.8, 879.1) | 1572.2 (1507.5, 1637) | 1452.1 (1386.6, 1517.6) |
| 2040 | 1031.5 (905.6, 1157.4) | 1601.7 (1385.3, 1818) | 1288.9 (1123, 1454.9) | 1002.1 (786.7, 1217.4) | 907.6 (787.4, 1027.8) | 1022.9 (765.6, 1280.2) | 838.4 (724.8, 952.1) | 1604.3 (1417.8, 1790.7) | 1413.1 (1236.3, 1589.8) |
| Observed Results: Percent change (1990 – 2021) | | | | | | | | | |
|  | -5.8% | -1.3% | 0.0% | -8.1% | -6.9% | -43.7% | -12.8% | 2.0% | -10.3% |
| Observed Results: Estimated Annual Percent change (1990 – 2021) | | | | | | | | | |
|  | -0.19% (-0.48%, 0.09%) | -0.04% (-0.26%, 0.17%) | 0.00% (-0.27%, 0.27%) | -0.27% (-0.55%, 0.01%) | -0.23% (-0.53%, 0.07%) | -1.84% (-2.06%, -1.61%) | -0.44% (-0.74%, -0.14%) | 0.06% (-0.16%, 0.29%) | -0.35% (-0.58%, -0.13%) |
| Projected Results: Percent change (2021 – 2040) | | | | | | | | | |
|  | 9.5% | -6.7% | 17.9% | 0.6% | 7.4% | -14.4% | -0.1% | 3.5% | -4.7% |
| Projected Results: Estimated Annual percent change (2021 – 2040) | | | | | | | | | |
|  | 0.48% (0.01%, 0.95%) | -0.36% (-0.72%, -0.01%) | 0.87% (0.44%, 1.3%) | 0.03% (-0.43%, 0.49%) | 0.37% (-0.12%, 0.87%) | -0.81% (-1.25%, -0.38%) | 0% (-0.51%, 0.5%) | 0.18% (-0.19%, 0.55%) | -0.25% (-0.64%, 0.13%) |

This table reports the age-standardized prevalence rates (per 100,000 individuals) of stroke among females in South Asia between 1990 and 2040. The age-standardized rates for the observed years (1990-2021) are reported with 95% confidence intervals. The age-standardized rates calculated using the Bayesian Age-Period-Cohort (BAPC) projections (2030, 2040) are reported as median value (25^th^ percentile, 75^th^ percentile). To evaluate the temporal change for each period we calculated the percent change between the initial and final value for that period. We further reported the estimated annual percentage change over the period. Confidence intervals for the estimated annual percentage change are calculated using bootstrap.

# Supplemental Table 11: Age-Standardized Stroke Mortality Rates

| Year | All South Asia | Afghanistan | Bangladesh | Bhutan | India | Maldives | Nepal | Pakistan | Sri Lanka |
| --- | --- | --- | --- | --- | --- | --- | --- | --- | --- |
| 1990 | 56.7 (56.4, 57.0) | 189.7 (184.3, 195.3) | 92.4 (90.9, 93.8) | 51.9 (38.5, 68.4) | 49.5 (49.2, 49.8) | 82.1 (55.0, 117.9) | 67.8 (65.2, 70.6) | 66.7 (65.6, 67.8) | 87.0 (84.2, 89.9) |
| 2000 | 53.2 (52.9, 53.4) | 204.9 (199.4, 210.4) | 77.0 (75.8, 78.2) | 45.4 (35.1, 57.8) | 46.5 (46.2, 46.7) | 65.2 (45.9, 89.6) | 55.4 (53.4, 57.5) | 79.4 (78.2, 80.5) | 81.4 (79.0, 83.8) |
| 2010 | 47.5 (47.3, 47.7) | 187.1 (181.9, 192.4) | 74.4 (73.5, 75.4) | 40.3 (31.9, 50.1) | 41.3 (41.1, 41.5) | 36.2 (24.6, 50.9) | 47.6 (46.1, 49.3) | 72.0 (71.0, 73.0) | 72.6 (70.7, 74.6) |
| 2021 | 44.9 (44.7, 45.0) | 167.7 (162.8, 172.8) | 65.2 (64.5, 65.9) | 38.2 (31.1, 46.4) | 39.7 (39.5, 39.9) | 27.8 (19.4, 38.6) | 44.4 (43.1, 45.7) | 68.3 (67.5, 69.1) | 54.8 (53.4, 56.1) |
| 2030 | 41.2 (37.1, 45.2) | 161.3 (147.8, 174.8) | 61.3 (54.4, 68.3) | 34.9 (29.7, 40.0) | 37.1 (32.8, 41.4) | 19.4 (15.6, 23.3) | 41.7 (38.1, 45.3) | 62.8 (57.1, 68.4) | 40.5 (33.8, 47.1) |
| 2040 | 38.2 (27.8, 48.5) | 162.1 (126.0, 198.1) | 57.2 (39.7, 74.7) | 32.8 (21.8, 43.8) | 34.1 (23.3, 44.9) | 14.2 (8.1, 20.4) | 38.2 (29.5, 46.9) | 58.7 (43.9, 73.6) | 29.8 (16.4, 43.1) |
| Observed Results: Percent change (1990 – 2021) | | | | | | | | | |
|  | -20.9% | -11.6% | -29.4% | -26.3% | -19.8% | -66.2% | -34.5% | 2.3% | -37.1% |
| Observed Results: Estimated Annual Percent change (1990 – 2021) | | | | | | | | | |
|  | -0.75% (-2%, 0.51%) | -0.4% (-1.06%, 0.27%) | -1.12% (-2.12%, -0.1%) | -0.98% (-2.31%, 0.36%) | -0.71% (-2.04%, 0.64%) | -3.43% (-4.76%, -2.08%) | -1.36% (-2.55%, -0.14%) | 0.08% (-1.01%, 1.17%) | -1.48% (-2.55%, -0.4%) |
| Projected Results: Percent change (2021 – 2040) | | | | | | | | | |
|  | -14.9% | -3.4% | -12.3% | -14.2% | -14.1% | -48.8% | -13.9% | -14.0% | -45.6% |
| Projected Results: Estimated Annual percent change (2021 – 2040) | | | | | | | | | |
|  | -0.85% (-3.07%, 1.43%) | -0.18% (-1.31%, 0.96%) | -0.69% (-2.53%, 1.19%) | -0.8% (-3.21%, 1.67%) | -0.8% (-3.16%, 1.62%) | -3.47% (-6.67%, -0.17%) | -0.79% (-3.02%, 1.5%) | -0.79% (-2.6%, 1.04%) | -3.16% (-5.4%, -0.85%) |

This table reports the age-standardized mortality rates (per 100,000 individuals) of stroke in South Asia between 1990 and 2040. The age-standardized rates for the observed years (1990-2021) are reported with 95% confidence intervals. The age-standardized rates calculated using the Bayesian Age-Period-Cohort (BAPC) projections (2030, 2040) are reported as median value (25^th^ percentile, 75^th^ percentile). To evaluate the temporal change for each period we calculated the percent change between the initial and final value for that period. We further reported the estimated annual percentage change over the period. Confidence intervals for the estimated annual percentage change are calculated using bootstrap.

# Supplemental Table 12. Male Age-Standardized Stroke Mortality Rates

| Year | All South Asia | Afghanistan | Bangladesh | Bhutan | India | Maldives | Nepal | Pakistan | Sri Lanka |
| --- | --- | --- | --- | --- | --- | --- | --- | --- | --- |
| 1990 | 57.8 (57.4, 58.3) | 204.4 (196.4, 212.6) | 97.2 (95.2, 99.2) | 39.9 (24.4, 61.6) | 50.3 (49.8, 50.7) | 87 (50.6, 139.3) | 57.8 (54.4, 61.4) | 64.6 (63.1, 66.1) | 106.6 (102.2, 111.1) |
| 2000 | 55.6 (55.2, 55.9) | 223.4 (215.2, 231.8) | 80.3 (78.6, 82) | 36.6 (24.5, 52.6) | 48.4 (48, 48.7) | 69.9 (43, 106.8) | 51.5 (48.7, 54.3) | 80.1 (78.5, 81.7) | 103.8 (99.9, 107.9) |
| 2010 | 51.6 (51.3, 51.9) | 208.6 (200.3, 217.1) | 77 (75.7, 78.3) | 38.4 (27.4, 52.1) | 44.9 (44.6, 45.2) | 50 (30.4, 76.8) | 48.9 (46.7, 51.3) | 76.5 (75, 77.9) | 92.5 (89.2, 95.8) |
| 2021 | 48.8 (48.6, 49.1) | 188.3 (179.9, 196.9) | 68.9 (68, 69.9) | 36.8 (27.3, 48.4) | 43.3 (43.1, 43.6) | 33.7 (20.7, 51.8) | 50.7 (48.7, 52.7) | 70.8 (69.6, 72) | 67.4 (65.2, 69.7) |
| 2030 | 48.9 (43.9, 53.8) | 185 (168.1, 202) | 66.9 (54.5, 79.3) | 35.6 (29.5, 41.8) | 44.9 (39.4, 50.4) | 21.3 (16.9, 25.7) | 49.4 (44.6, 54.2) | 65.9 (60.1, 71.8) | 48.3 (39.1, 57.6) |
| 2040 | 49.6 (35.9, 63.3) | 195 (148.7, 241.2) | 65.8 (31.8, 99.7) | 34.4 (21.3, 47.5) | 46.2 (30.8, 61.5) | 13.2 (7.6, 18.9) | 47.1 (35.2, 58.9) | 62.8 (47.4, 78.1) | 34.1 (16.2, 52) |
| Observed Results: Percent change (1990 – 2021) | | | | | | | | | |
|  | -15.6% | -7.9% | -29.1% | -7.8% | -13.8% | -61.2% | -12.3% | 9.7% | -36.7% |
| Observed Results: Estimated Annual Percent change (1990 – 2021) | | | | | | | | | |
|  | -0.54% (-1.76%, 0.69%) | -0.26% (-0.90%, 0.37%) | -1.10% (-2.08%, -0.11%) | -0.26% (-1.69%, 1.19%) | -0.48% (-1.78%, 0.83%) | -3.01% (-4.25%, -1.76%) | -0.42% (-1.63%, 0.80%) | 0.30% (-0.79%, 1.39%) | -1.47% (-2.43%, -0.49%) |
| Projected Results: Percent change (2021 – 2040) | | | | | | | | | |
|  | 1.7% | 3.5% | -4.6% | -6.5% | 6.5% | -60.7% | -7.2% | -11.4% | -49.5% |
| Projected Results: Estimated Annual percent change (2021 – 2040) | | | | | | | | | |
|  | 0.09% (-1.97%, 2.19%) | 0.18% (-0.87%, 1.25%) | -0.24% (-2%, 1.55%) | -0.35% (-2.76%, 2.11%) | 0.34% (-1.82%, 2.56%) | -4.81% (-7.95%, -1.57%) | -0.39% (-2.44%, 1.71%) | -0.63% (-2.39%, 1.16%) | -3.52% (-5.59%, -1.41%) |

This table reports the age-standardized mortality rates (per 100,000 individuals) of stroke among males in South Asia between 1990 and 2040. The age-standardized rates for the observed years (1990-2021) are reported with 95% confidence intervals. The age-standardized rates calculated using the Bayesian Age-Period-Cohort (BAPC) projections (2030, 2040) are reported as median value (25^th^ percentile, 75^th^ percentile). To evaluate the temporal change for each period we calculated the percent change between the initial and final value for that period. We further reported the estimated annual percentage change over the period. Confidence intervals for the estimated annual percentage change are calculated using bootstrap.

# Supplemental Table 13. Female Age-Standardized Stroke Mortality Rates

| Year | All South Asia | Afghanistan | Bangladesh | Bhutan | India | Maldives | Nepal | Pakistan | Sri Lanka |
| --- | --- | --- | --- | --- | --- | --- | --- | --- | --- |
| 1990 | 55.5 (55.1, 56) | 175.1 (167.7, 182.7) | 86.6 (84.5, 88.7) | 64.9 (43.8, 92.6) | 48.7 (48.2, 49.2) | 82.4 (43.8, 140.9) | 78.5 (74.4, 82.7) | 69.1 (67.5, 70.8) | 67.2 (63.6, 70.8) |
| 2000 | 50.8 (50.5, 51.2) | 186.2 (179.1, 193.6) | 73 (71.3, 74.8) | 54.3 (37.9, 75.2) | 44.7 (44.3, 45.1) | 54.4 (30.9, 88.7) | 59.5 (56.5, 62.7) | 78.1 (76.5, 79.8) | 61.3 (58.5, 64.3) |
| 2010 | 43.5 (43.2, 43.7) | 166.9 (160.4, 173.6) | 71.2 (69.9, 72.5) | 42.3 (30.1, 57.7) | 37.8 (37.6, 38.1) | 27.3 (14.5, 46.1) | 46.4 (44.2, 48.7) | 66.6 (65.3, 68) | 56.8 (54.5, 59.1) |
| 2021 | 41.1 (40.8, 41.3) | 150.4 (144.5, 156.5) | 61 (60.1, 62) | 39.4 (29.2, 52) | 36.2 (36, 36.5) | 20.1 (10.8, 34) | 38.9 (37.2, 40.6) | 64.6 (63.6, 65.8) | 44.7 (43.1, 46.3) |
| 2030 | 35.3 (31.4, 39.3) | 144.4 (131.6, 157.3) | 55.3 (50.6, 59.9) | 32.7 (26.4, 38.9) | 31.3 (27, 35.6) | 9.1 (6.5, 11.7) | 35.2 (31.7, 38.6) | 58.5 (52, 65.1) | 34.2 (30.8, 37.6) |
| 2040 | 30.6 (21.3, 39.9) | 144.9 (111.3, 178.5) | 49.1 (38.6, 59.5) | 27.3 (15.9, 38.6) | 26.4 (16.6, 36.1) | 4.2 (1.6, 6.7) | 31.7 (23.7, 39.8) | 53.7 (37, 70.4) | 26.2 (19.5, 33) |
| Observed Results: Percent change (1990 – 2021) | | | | | | | | | |
|  | -26.0% | -14.1% | -29.5% | -39.2% | -25.6% | -75.7% | -50.5% | -6.5% | -33.4% |
| Observed Results: Estimated Annual Percent change (1990 – 2021) | | | | | | | | | |
|  | -0.96% (-2.24%, 0.33%) | -0.49% (-1.19%, 0.21%) | -1.12% (-2.16%, -0.07%) | -1.6% (-2.85%, -0.33%) | -0.95% (-2.32%, 0.43%) | -4.45% (-5.94%, -2.93%) | -2.24% (-3.44%, -1.02%) | -0.22% (-1.3%, 0.88%) | -1.31% (-2.5%, -0.09%) |
| Projected Results: Percent change (2021 – 2040) | | | | | | | | | |
|  | -25.5% | -3.7% | -19.6% | -30.9% | -27.2% | -79.2% | -18.3% | -16.9% | -41.3% |
| Projected Results: Estimated Annual percent change (2021 – 2040) | | | | | | | | | |
|  | -1.54% (-3.94%, 0.91%) | -0.2% (-1.39%, 1.01%) | -1.14% (-3.07%, 0.84%) | -1.91% (-4.4%, 0.64%) | -1.65% (-4.21%, 0.98%) | -7.91% (-12.87%, -2.67%) | -1.07% (-3.48%, 1.4%) | -0.97% (-2.84%, 0.94%) | -2.77% (-5.21%, -0.27%) |

This table reports the age-standardized mortality rates (per 100,000 individuals) of stroke among females in South Asia between 1990 and 2040. The age-standardized rates for the observed years (1990-2021) are reported with 95% confidence intervals. The age-standardized rates calculated using the Bayesian Age-Period-Cohort (BAPC) projections (2030, 2040) are reported as median value (25^th^ percentile, 75^th^ percentile). To evaluate the temporal change for each period we calculated the percent change between the initial and final value for that period. We further reported the estimated annual percentage change over the period. Confidence intervals for the estimated annual percentage change are calculated using bootstrap.

# Supplemental Table 14: Age-Standardized PAD Prevalence Rates

| Year | All South Asia | Afghanistan | Bangladesh | Bhutan | India | Maldives | Nepal | Pakistan | Sri Lanka |
| --- | --- | --- | --- | --- | --- | --- | --- | --- | --- |
| 1990 | 1670.2 (1668.5, 1671.8) | 1594.7 (1578.8, 1610.7) | 1552.7 (1546.8, 1558.6) | 1373.7 (1300.7, 1449.7) | 1635.8 (1633.9, 1637.7) | 2644.4 (2477.5, 2819.6) | 1611.4 (1598.4, 1624.5) | 1891.4 (1885.4, 1897.4) | 2688.9 (2673.0, 2704.8) |
| 2000 | 1750.3 (1748.8, 1751.8) | 1693.8 (1677.7, 1710.0) | 1553.5 (1548.3, 1558.8) | 1411.8 (1348.3, 1477.4) | 1719.2 (1717.6, 1720.9) | 2790.6 (2644.6, 2942.3) | 1751.6 (1739.8, 1763.5) | 1956.5 (1950.9, 1962.1) | 2885.7 (2871.5, 2900.1) |
| 2010 | 1786.4 (1785.2, 1787.7) | 1909.2 (1891.7, 1926.9) | 1549.7 (1545.5, 1553.9) | 1478.2 (1421.2, 1536.8) | 1750.1 (1748.7, 1751.5) | 2859.2 (2734.2, 2988.2) | 1787.5 (1777.4, 1797.7) | 2054.9 (2049.8, 2060.0) | 3119.4 (3106.7, 3132.2) |
| 2021 | 1809.5 (1808.5, 1810.6) | 2128.9 (2111.2, 2146.7) | 1646.1 (1642.6, 1649.5) | 1507.0 (1457.1, 1558.2) | 1773.5 (1772.3, 1774.7) | 2807.1 (2714.9, 2901.6) | 1733.5 (1724.9, 1742.1) | 2024.8 (2020.5, 2029.0) | 3133.9 (3123.0, 3144.8) |
| 2030 | 1842.1 (1774.9, 1909.4) | 2193.5 (2089.7, 2297.3) | 1688.4 (1620.7, 1756.1) | 1538.1 (1431.1, 1645.1) | 1805.4 (1737.5, 1873.3) | 2762.5 (2568.1, 2957.0) | 1772.7 (1687.4, 1858.1) | 2072.0 (1986.7, 2157.3) | 3132.7 (3003.3, 3262.0) |
| 2040 | 1879.5 (1684.9, 2074.0) | 2233.0 (1941.5, 2524.5) | 1731.5 (1535.2, 1927.8) | 1570.7 (1287.0, 1854.5) | 1840.3 (1644.0, 2036.6) | 2684.3 (2193.5, 3175.2) | 1826.5 (1579.9, 2073.1) | 2135.5 (1887.3, 2383.7) | 3130.6 (2767.6, 3493.6) |
| Observed Results: Percent change (1990 – 2021) | | | | | | | | | |
|  | 8.3% | 33.5% | 6.0% | 9.7% | 8.4% | 6.2% | 7.6% | 7.1% | 16.6% |
| Observed Results: Estimated Annual Percent change (1990 – 2021) | | | | | | | | | |
|  | 0.26% (0.04%, 0.47%) | 0.94% (0.73%, 1.15%) | 0.19% (-0.04%, 0.41%) | 0.3% (0.06%, 0.54%) | 0.26% (0.04%, 0.48%) | 0.19% (0.02%, 0.36%) | 0.24% (0.02%, 0.46%) | 0.22% (0.02%, 0.42%) | 0.5% (0.33%, 0.66%) |
| Projected Results: Percent change (2021 – 2040) | | | | | | | | | |
|  | 3.9% | 4.9% | 5.2% | 4.2% | 3.8% | -4.4% | 5.4% | 5.5% | -0.1% |
| Projected Results: Estimated Annual percent change (2021 – 2040) | | | | | | | | | |
|  | 0.2% (-0.14%, 0.54%) | 0.25% (-0.06%, 0.57%) | 0.27% (-0.09%, 0.62%) | 0.22% (-0.15%, 0.59%) | 0.19% (-0.15%, 0.54%) | -0.24% (-0.51%, 0.04%) | 0.28% (-0.07%, 0.62%) | 0.28% (-0.04%, 0.6%) | -0.01% (-0.27%, 0.26%) |

This table reports the age-standardized prevalence rates (per 100,000 individuals) of peripheral artery disease (PAD) in South Asia between 1990 and 2040. The age-standardized rates for the observed years (1990-2021) are reported with 95% confidence intervals. The age-standardized rates calculated using the Bayesian Age-Period-Cohort (BAPC) projections (2030, 2040) are reported as median value (25^th^ percentile, 75^th^ percentile). To evaluate the temporal change for each period we calculated the percent change between the initial and final value for that period. We further reported the estimated annual percentage change over the period. Confidence intervals for the estimated annual percentage change are calculated using bootstrap.

# Supplemental Table 15. Male Age-Standardized PAD Prevalence Rates

| Year | All South Asia | Afghanistan | Bangladesh | Bhutan | India | Maldives | Nepal | Pakistan | Sri Lanka |
| --- | --- | --- | --- | --- | --- | --- | --- | --- | --- |
| 1990 | 1230.7 (1228.8, 1232.7) | 1419.4 (1398.2, 1440.9) | 1337.8 (1330.4, 1345.1) | 1124.8 (1033.9, 1221.6) | 1174.4 (1172.2, 1176.5) | 2281.8 (2074.9, 2503.7) | 1360.5 (1343.9, 1377.3) | 1422 (1414.9, 1429.1) | 2117 (2097.1, 2137) |
| 2000 | 1273 (1271.2, 1274.8) | 1510.6 (1488.4, 1533) | 1340.7 (1334.1, 1347.4) | 1162.9 (1084.3, 1245.7) | 1219.1 (1217.2, 1221.1) | 2395.8 (2210.6, 2592) | 1468.4 (1453.2, 1483.7) | 1443 (1436.3, 1449.8) | 2277.8 (2259.4, 2296.3) |
| 2010 | 1321.2 (1319.6, 1322.7) | 1686.7 (1661.5, 1712.1) | 1338.3 (1333, 1343.7) | 1226.4 (1155.2, 1300.8) | 1267 (1265.3, 1268.7) | 2433.7 (2271.8, 2603.6) | 1505.6 (1492.4, 1518.9) | 1521.8 (1515.6, 1528) | 2427.3 (2410.7, 2444) |
| 2021 | 1334.4 (1333.1, 1335.7) | 1864.6 (1838.1, 1891.4) | 1401.2 (1396.7, 1405.7) | 1243.1 (1180.3, 1308.4) | 1277 (1275.6, 1278.5) | 2399.8 (2281.9, 2522.1) | 1443.4 (1432, 1454.9) | 1522.1 (1516.8, 1527.3) | 2431.7 (2417.5, 2446) |
| 2030 | 1329.7 (1277.5, 1381.8) | 1923.6 (1822.5, 2024.8) | 1396.3 (1338.3, 1454.2) | 1254.6 (1153.5, 1355.8) | 1271.9 (1219.1, 1324.7) | 2413 (2216.1, 2610) | 1447.4 (1372.5, 1522.2) | 1526.1 (1463.9, 1588.3) | 2462.5 (2350.9, 2574.1) |
| 2040 | 1318.9 (1172, 1465.7) | 1976.5 (1694.6, 2258.4) | 1384.2 (1222.8, 1545.6) | 1261.5 (1003.9, 1519.1) | 1261.2 (1112.4, 1409.9) | 2422.6 (1921, 2924.2) | 1452.8 (1244.3, 1661.2) | 1528.1 (1353.5, 1702.7) | 2495.3 (2180.4, 2810.3) |
| Observed Results: Percent change (1990 – 2021) | | | | | | | | | |
|  | 8.4% | 31.4% | 4.7% | 10.5% | 8.7% | 5.2% | 6.1% | 7% | 14.9% |
| Observed Results: Estimated Annual Percent change (1990 – 2021) | | | | | | | | | |
|  | 0.26% (0.01%, 0.51%) | 0.88% (0.66%, 1.11%) | 0.15% (-0.09%, 0.39%) | 0.32% (0.06%, 0.58%) | 0.27% (0.01%, 0.53%) | 0.16% (-0.02%, 0.35%) | 0.19% (-0.05%, 0.43%) | 0.22% (-0.01%, 0.45%) | 0.45% (0.26%, 0.64%) |
| Projected Results: Percent change (2021 – 2040) | | | | | | | | | |
|  | -1.2% | 6% | -1.2% | 1.5% | -1.2% | 0.9% | 0.6% | 0.4% | 2.6% |
| Projected Results: Estimated Annual percent change (2021 – 2040) | | | | | | | | | |
|  | -0.06% (-0.46%, 0.34%) | 0.31% (-0.03%, 0.64%) | -0.06% (-0.45%, 0.33%) | 0.08% (-0.33%, 0.49%) | -0.07% (-0.47%, 0.34%) | 0.05% (-0.25%, 0.35%) | 0.03% (-0.35%, 0.42%) | 0.02% (-0.35%, 0.4%) | 0.14% (-0.16%, 0.43%) |

This table reports the age-standardized prevalence rates (per 100,000 individuals) of peripheral artery disease (PAD) among males in South Asia between 1990 and 2040. The age-standardized rates for the observed years (1990-2021) are reported with 95% confidence intervals. The age-standardized rates calculated using the Bayesian Age-Period-Cohort (BAPC) projections (2030, 2040) are reported as median value (25^th^ percentile, 75^th^ percentile). To evaluate the temporal change for each period we calculated the percent change between the initial and final value for that period. We further reported the estimated annual percentage change over the period. Confidence intervals for the estimated annual percentage change are calculated using bootstrap.

# Supplemental Table 16. Female Age-Standardized PAD Prevalence Rates

| Year | All South Asia | Afghanistan | Bangladesh | Bhutan | India | Maldives | Nepal | Pakistan | Sri Lanka |
| --- | --- | --- | --- | --- | --- | --- | --- | --- | --- |
| 1990 | 2159.9 (2157.1, 2162.7) | 1768.2 (1744.7, 1792) | 1810.4 (1801, 1819.8) | 1643.6 (1528.8, 1764.7) | 2148.3 (2145.2, 2151.4) | 3106 (2837, 3393.6) | 1875.8 (1855.7, 1896) | 2422.1 (2412.2, 2431.9) | 3265.6 (3240.8, 3290.5) |
| 2000 | 2255.7 (2253.3, 2258.2) | 1844.1 (1821.1, 1867.2) | 1813.2 (1804.7, 1821.7) | 1701.3 (1599.7, 1807.5) | 2248.6 (2245.9, 2251.3) | 3196.4 (2971, 3433.9) | 2044.8 (2026.6, 2063.2) | 2493.5 (2484.5, 2502.6) | 3434 (3412.5, 3455.6) |
| 2010 | 2253.8 (2251.8, 2255.9) | 2050.9 (2027, 2075) | 1784 (1777.5, 1790.6) | 1771.3 (1680.9, 1865.3) | 2237.5 (2235.2, 2239.8) | 3249.6 (3062.1, 3445) | 2065.9 (2050.6, 2081.4) | 2584.4 (2576.3, 2592.5) | 3693.7 (3674.9, 3712.5) |
| 2021 | 2278.9 (2277.1, 2280.6) | 2274.4 (2251.3, 2297.6) | 1888.9 (1883.7, 1894.2) | 1798.1 (1719.7, 1879) | 2270.3 (2268.4, 2272.3) | 3233.9 (3089.9, 3382.7) | 1987.8 (1975.2, 2000.4) | 2507.3 (2500.7, 2513.9) | 3709.9 (3693.8, 3726) |
| 2030 | 2334.7 (2248.4, 2420.9) | 2360.8 (2242.1, 2479.5) | 1948.7 (1863.7, 2033.8) | 1832.3 (1690.9, 1973.7) | 2331.1 (2243.1, 2419) | 3232.9 (2979.3, 3486.6) | 2021.7 (1919.6, 2123.7) | 2562.9 (2449, 2676.8) | 3689.9 (3532.2, 3847.7) |
| 2040 | 2408.2 (2156, 2660.3) | 2441.8 (2105.1, 2778.5) | 2015.6 (1767.4, 2263.7) | 1864.1 (1497.1, 2231) | 2407.2 (2149.6, 2664.8) | 3223.6 (2576.2, 3870.9) | 2071.2 (1779.6, 2362.8) | 2645.5 (2313.9, 2977.1) | 3669.2 (3230.2, 4108.1) |
| Observed Results: Percent change (1990 – 2021) | | | | | | | | | |
|  | 5.5% | 28.6% | 4.3% | 9.4% | 5.7% | 4.1% | 6% | 3.5% | 13.6% |
| Observed Results: Estimated Annual Percent change (1990 – 2021) | | | | | | | | | |
|  | 0.17% (-0.02%, 0.36%) | 0.82% (0.61%, 1.02%) | 0.14% (-0.07%, 0.35%) | 0.29% (0.07%, 0.51%) | 0.18% (-0.01%, 0.37%) | 0.13% (-0.03%, 0.29%) | 0.19% (-0.02%, 0.39%) | 0.11% (-0.07%, 0.29%) | 0.41% (0.26%, 0.56%) |
| Projected Results: Percent change (2021 – 2040) | | | | | | | | | |
|  | 5.7% | 7.4% | 6.7% | 3.7% | 6% | -0.3% | 4.2% | 5.5% | -1.1% |
| Projected Results: Estimated Annual percent change (2021 – 2040) | | | | | | | | | |
|  | 0.29% (-0.01%, 0.59%) | 0.37% (0.07%, 0.68%) | 0.34% (0.01%, 0.67%) | 0.19% (-0.15%, 0.53%) | 0.31% (0.01%, 0.61%) | -0.02% (-0.27%, 0.24%) | 0.22% (-0.11%, 0.54%) | 0.28% (-0.01%, 0.57%) | -0.06% (-0.3%, 0.18%) |

This table reports the age-standardized prevalence rates (per 100,000 individuals) of peripheral artery disease (PAD) among females in South Asia between 1990 and 2040. The age-standardized rates for the observed years (1990-2021) are reported with 95% confidence intervals. The age-standardized rates calculated using the Bayesian Age-Period-Cohort (BAPC) projections (2030, 2040) are reported as median value (25^th^ percentile, 75^th^ percentile). To evaluate the temporal change for each period we calculated the percent change between the initial and final value for that period. We further reported the estimated annual percentage change over the period. Confidence intervals for the estimated annual percentage change are calculated using bootstrap.

# Supplemental Statistical Methods

Bayesian Age Period Cohort Model.

Age-Period-Cohort models are recommended by demographers as the correct method to evaluate temporal changes in data & project future rates. They suffer from an identifiability problem as there is an exact linear relationship between age, period, and cohort as one can be directly calculated from the other two. Due to this linear dependence, it is impossible to identify separate contributions of the age, period and cohort effects. The observed event rate for the data can be presented as:

$$log(\lambda_{ij})= \alpha(age - i) + \beta(period - j) + \gamma(cohort ) + \mu(intercept term)$$

While the identifiability problem can make it challenging to identify individual contributions of age, period and cohort, accurately projecting future rates is more easily possible. However, in this study, our primary aim was to be able to reliably project future event rates, which can be reliably identified using these models. The Bayesian age-period-cohort models also model non-linear changes in the observed data. However, unlike, frequentist approaches, where spline terms need to be fitted with predetermined knots or polynomial terms need to be chosen a-priori, this model does not need such inputs from the user. Hence, it chooses an equation that is best suited to the data.

Model priors: We used smoothing priors for the age, period and cohort effects. The standard choice is to use the second-order random walk (RW2) which assumes independent mean-zero normal distributions (with unknown variance) on the second differences of all time effects. The RW2 smoothing prior can be presented as: Δ²θᵢ = θᵢ - 2θᵢ₋₁ + θᵢ₋₂ ~ N(0, σ²) where θ represents the parameter for the i-th level. The benefits of using the RW2 prior are: 1. It helps smooth the estimates effects across adjacent periods and reduces the noise and improves interpretability. 2. It breaks the perfect collinearity between the three effects and therefore all three effects can be simultaneously introduced into the model.

Model convergence and diagnostic accuracy: We used the Integrated Network Laplace Approximation (INLA) method to approximate the posterior marginal distributions directly from the model without Markov Chain Monte Carlo (MCMC) sampling method. The INLA approach is faster, does not have any convergence concerns, and provides very similar results to the traditional MCMC approach. The model’s predictive accuracy was checked by calculating the Brier score, root mean square error and mean absolute error from the observed and model predicted values between 1990 - 2021. Additionally, we graphed and compared the observed and predicted estimates between 1990 -2021 for concordance.

We refer readers to the following manuscripts for further information regarding the BAPC package and fitting BAPC models using INLA in R:

1. <https://rdrr.io/rforge/BAPC/> - this webpage provides information regarding the functions present in the BAPC package.

2. Riebler A, Held L. Projecting the future burden of cancer: Bayesian age-period-cohort analysis with integrated nested Laplace approximations. Biom J. 2017 May;59(3):531-549. doi: 10.1002/bimj.201500263. Epub 2017 Jan 31. PMID: 28139001. – This is their manuscript wherein they present the details of their modeling process.

3. Supplemental material to the above paper provides R code used for the examples presented in their manuscript.

4. <https://cran.r-project.org/web/packages/scoringRules/index.html> - We used the scoringRules R package to calculate the model metrics
